# Supplementary material for: Defective glutamate and K+ clearance by cortical astrocytes in familial hemiplegic migraine type 2
Source: EMBO Mol Med. 2016 Jun 27;8(8):967–86. doi: 10.15252/emmm.201505944 (PMC4967947; doi:10.15252/emmm.201505944)
Supplement: Supplementary file 1 — Appendix [file EMMM-8-967-s001.pdf]

Table of content

Appendix Figure S1

Appendix Figure S2

Appendix Figure legends

Appendix Discussion

Appendix Materials and Methods

Appendix References

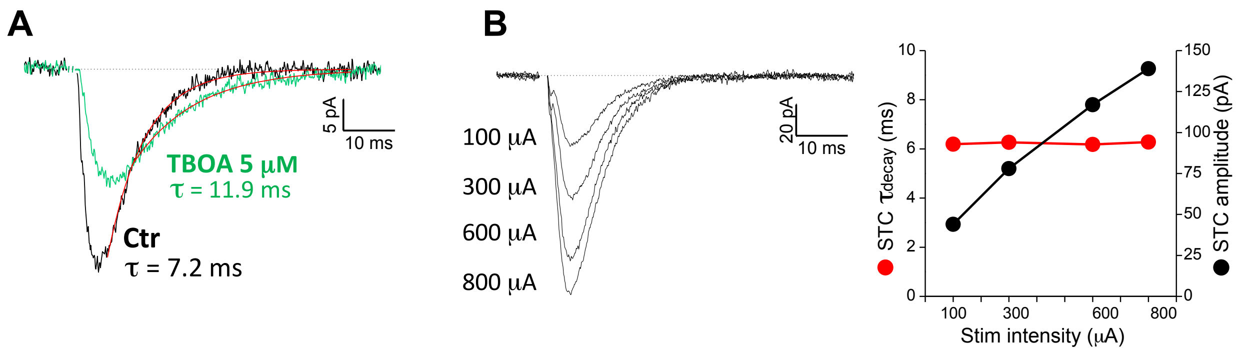

Appendix Figure S1

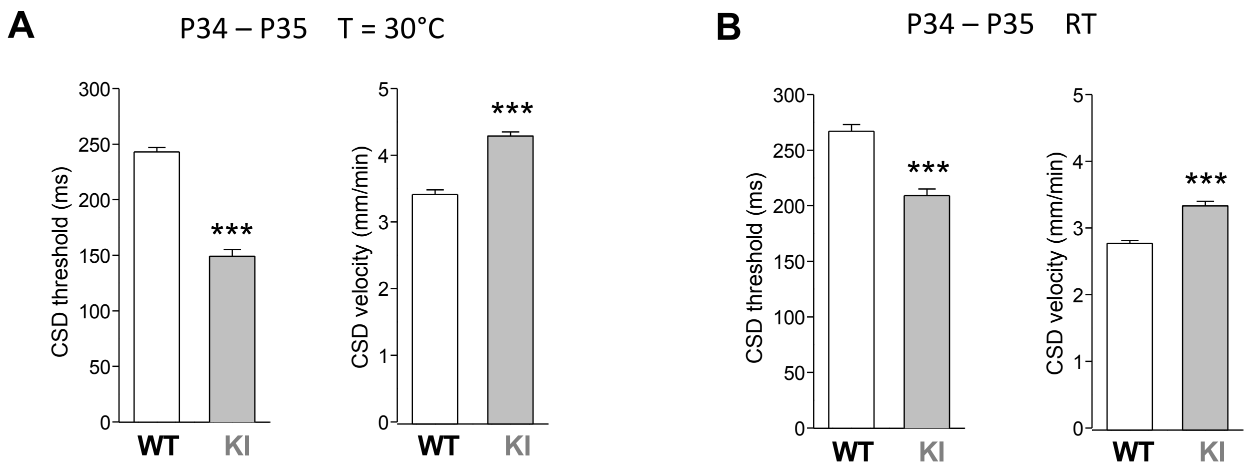

Appendix Figure S2

Appendix figure legends

Figure S1. The decay kinetics of the STC reflect the rate of glutamate clearance by astrocytes and provide a measure of the rate of glutamate clearance which is independent of the amount of glutamate released.

(A) Inhibition of a fraction of glutamate transporters with DL-TBOA increases τ_{decay} of the STC elicited by single pulse stimulation in layer 1 astrocytes in WT cortical slices. In the representative experiment shown here, 5 µM DL-TBOA increased by 65% τ_{decay} of the STC (from 6.6 ms to 12.6 ms) and decreased the STC amplitude by 42%. The STC was isolated pharmacologically by subtracting the current recorded in the presence of saturating concentrations of DL-TBOA (100 µM) from the total control current and the total current recorded in the presence of 5 µM DL-TBOA (see Fig 1B of main text). P23 (n=3; N=2).

(B) Increasing the intensity of the extracellular stimulation increases the amplitude without affecting the decay kinetics of the STC elicited by single pulse stimulation in layer 1

astrocytes in WT cortical slice. STC traces at increasing intensity of extracellular stimulation (left) together with τ_{decay} and amplitude of the STC as a function of the intensity of stimulation (right) in a representative experiment in which the stimulation intensity was increased from 100 to 800 µA. The STC was isolated by subtracting from the total inward current an exponential waveform that approximates the average TBOA-insensitive current (see Fig 1C of main text). P21 (n=3; N=3).

Figure S2. Facilitation of CSD induction and propagation in acute cortical slices of P34-35 W887R/+ FHM2 KI mice at different temperatures.

(A) CSD threshold and CSD velocity in WT (n = 20; N=3) and FHM2 KI (n = 26; N=4) cortical slices from P34-35 mice (T = 30 °C, flow rate 13 ml/min). CSD threshold is 39% lower in FHM2 KI relative to WT mice (149 ± 6 vs 243 ± 4 ms; Mann-Whitney U test, P < 0.0001). CSD velocity is 26% higher in FHM2 KI relative to WT mice (4.29 ± 0.06 vs 3.41 ± 0.07 mm/min; unpaired t test, P < 0.0001).

(B) CSD threshold and CSD velocity in WT (n = 15; N=2) and FHM2 KI (n = 17; N=2) cortical slices from P34-35 mice (room temperature, flow rate 3 ml/min). CSD threshold is 22% lower in FHM2 KI relative to WT mice (209 ± 6 vs 267 ± 6 ms; Mann-Whitney U test, P < 0.0001). CSD velocity is 20% higher in FHM2 KI compared to WT mice (3.33 ± 0.07 vs 2.77 ± 0.04 mm/min; unpaired t test: P < 0.0001).

Appendix Discussion

A slowing of the Na^+-$HCO3^-$ cotransport is expected to increase activity-dependent alkaline shifts and, as a consequence, possibly lead to increased activation of NMDARs (CHESLER, 2003), thus providing an additional mechanism that could contribute to facilitate CSD in FHM2 KI mice. Given that the Na^+-Ca^{2+} exchanger (NCX) reversal potential lies close to the resting membrane potential, reduced expression of the α_2 NKA might favor the reverse mode of the NCX in FHM2 astrocytes, which could generate local Ca^{2+} increases in perisynaptic processes (where NCX is prominently localized: (Minelli et al, 2007)) and in the microdomains at the plasma membrane-endoplasmic reticulum (ER) junctions (where NCX colocalizes with α_2 NKA in cultured astrocytes: (Juhaszova & Blaustein, 1997)), hence increasing ER Ca content; this could result in increased release of gliotransmitters, including Glu, that might be involved in CSD facilitation. Interestingly, evidence for increased Ca^{2+} in the ER of astrocytes cultured from α2 NKA knockout mice has been reported (Golovina et al, 2003).

Finally, considering that the α2 NKA appears expressed primarily in neurons during embryonic development (Ikeda et al, 2003; Moseley et al, 2003), possible alterations of cortical network development produced by the FHM2 mutation and consequent effects on cortical synaptic function might be additional hypothetical mechanisms contributing to CSD facilitation in FHM2 KI mice.

However, as discussed in the main text, if present, the contribution of mechanisms involving Na+- dependent transporters (or deriving from hypothetical alterations of cortical network development) to the facilitation of CSD initiation is small and one should assume that they may contribute more to facilitation of CSD propagation.

Appendix Materials and Methods

Acute slices preparation

Acute coronal slices of somatosensory cortex were prepared from wild-type (WT) and heterozygous W887R/+ knockin (KI) mice from the same litter as described in (Tottene et al, 2009). Briefly, animals were anestethized and decapitated. The brain was quickly removed and put in an ice-cold cutting solution (in mM: 130 K gluconate, 15 KCl, 0.2 EGTA, 20 HEPES, 25 glucose, 2 kynurenic acid, 5 x 10^{-5} minocycline, pH 7.4 with NaOH, oxygenated with 100% O_2) (Dugue et al, 2005). 350 μm-thick slices were then cut on the coronal plane with a vibratome (VT1200S, Leica Biosystems, Germany) and were transferred for 1 min in a solution containing (in mM) 225 D-mannitol, 2.5 KCl, 1.25 NaH_2PO_4, 26 $NaHCO_3$, 25 glucose, 0.8 $CaCl_2$, 8 $MgCl_2$, 2 kynurenic acid, 5 x 10^{-5} minocycline, saturated with 95% O_2 and 5% CO_2. Slices were then maintained at 30 °C for 30 min in standard artificial cerebrospinal fluid saturated with 95% O_2 and 5% CO_2 (sACSF in mM: 125 NaCl, 2.5 KCl, 25 $NaHCO_3$, 1.25 NaH_2PO_4, 1 $MgCl_2$, 2 $CaCl_2$, 25 glucose) plus 50 nM minocycline, and then transferred at room temperature in the same solution for a minimum of 30 min.

Acute cortical slices prepared as described above were used for measurement of either the current elicited in layer 1 astrocytes by extracellular stimulation (comparison WT vs KI) or of threshold and velocity of cortical spreading depression induced by high KCl pulses (comparison WT vs KI or ceftriaxone-treated vs untreated). In most CSD measurements (including all those testing the ceftriaxone effect) the investigator was blinded to the genotype. The investigator was not blinded to the genotype in the astrocyte current measurements.

Cortical spreading depression

Cortical spreading depression was elicited and measured in acute coronal slices of the somatosensory cortex of WT and FHM2 KI mice as in (Tottene et al, 2009), but most recordings were at 30°C rather than room temperature and the rate of perfusion of the slices was higher. Briefly, the brain slices were placed into a submersion chamber and continuously perfused with fresh extracellular solution (as that used for astrocyte patch-clamp recordings but with 3.5 mM KCl) at 30 °C at a flow rate of 6 mL/min (unless otherwise specified), and pressure-ejection pulses of 3 M KCl (0.5 bar) of increasing duration (at 5 min intervals in 20 ms steps) were applied through a glass micropipette (R = 0.19-0.25 MΩ) onto the slice surface on layer 2/3, using a PDES-02DX pneumatic drug ejection system (Npi Electronic GmbH, Tamm, Germany), until a CSD was elicited. CSD was detected by monitoring the associated change in intrinsic optical signal (IOS). The duration of the first pulse eliciting a CSD was taken as CSD threshold and the rate of horizontal spread of the change in IOS as CSD velocity. IOS was recorded using a CCD camera (KP-M2A, Hitachi, Tokio, Japan) connected with an upright microscope (Nikon Eclipse; 10X magnification, Nikon, Tokio, Japan). Images were recorded at 200 ms intervals as 640 x 480 pixels images (pixel size: 2.15 μm). MBF ImageJ software was used for the off line analysis of the digitalized images. The IOS change is expressed as percent change in light trasmittance (ΔT) relative to the background signal: %ΔT/T.

All CSD experiments and analyses in saline-treated and cef-treated mice and most of the CSD experiments in WT and KI mice were performed by a blinded observer.

Immunocytochemical studies

Immunofluorescence. After washing in PB, sections to be used for GLT-1/VGLUT1 double-labeling studies were directly incubated in 10% normal goat serum (NGS) in PB (1 h), whereas those to be used for GLT-1a/α_2/VGLUT1 triple-labeling studies were pre-treated

with 10%, 20% and 10% alcohol in PB (5 min each) and then incubated in NGS. Then, sections were exposed (2 h at RT and then overnight at 4 C°) to a solution containing a mixture of antibodies directed against the synthetic peptide corresponding to AA 559-573 (SADCSVEEEPWKREK) of GLT-1a rat C-terminus (0.3 μg/mL; made in rabbit) kindly provided by Dr J.D. Rothstein, Johns Hopkins University, Baltimore, MD (Omrani et al, 2009; Rothstein et al, 1994) and raised against a C-terminus synthetic peptide of rat VGLUT1 protein (GATHSTVQPPRPPPPVRDY) (1:800; made in guinea pig; AB5905, Merck Millipore, Billerica, MA; Melone et al., 2005) for double-labeling studies, or to a solution containing a mixture of antibodies direct against a synthetic peptide corresponding to AA 554–573 (AANGKSADCSVEEEPWKREK) of GLT-1a rat C-terminus (1: 250; made in guinea pig, AB1783, Merck Millipore; (DeSilva et al, 2012)), against a synthetic peptide corresponding to AA 432-445 of human α_2 Na^+,K^+ ATPase (CKAGQENISVSKRDT; immunizing sequence is identical in rat and mouse; 1:200, made in rabbit; AB07-674, Merck Millipore), and against Strep-Tag® fusion protein of the rat VGLUT1 (AA 456-560), specific for rat and mouse VGLUT1 (1:50, made in mouse; Synaptic System, Goettingen, Germany) for triple-labeling studies. Sections were washed and incubated in 10% NGS in PB (15 min) and then in a solution containing a mixture of affinity-purified Alexa 488- (1:250; 111-545-003, Jackson ImmunoResearch Europe, Ltd, Suffolk, UK) or Alexa 555- (1:250; TRITC, T-2762/6691-11:250; Molecular Probes, PoortGebouw, The Netherlands)-conjugated secondary antibodies made in goats (90 min) for double-labeling studies or Alexa 488-555- and 647-(1:200, Jackson) conjugated secondary antibodies made in goats for triple-labeling studies. Sections were washed, mounted, air-dried, and coverslipped using Vectashield mounting medium (H-1000; Jackson), and finally examined with a Leica confocal laser microscope (TCS SP2; Leica Microsystems, Wetzlar, Germany). Control experiments with single-labeled sections and sections incubated with two or three primary antibodies and one secondary antibody or with one primary and two or three secondary

antibodies revealed neither bleed-through nor cross-reactivity.

Confocal microscopy and data analysis. Microscopic fields from layers II/III of SI were randomly collected. Analysis of positive (+) puncta (i.e. GLT-1a, VGLUT1+ puncta in double-labeled material and GLT-1a, α_2, VGLUT1+ puncta in triple-labeled material) was performed in randomly selected subfields of 20 × 20 µm. Images of selected subfields were processed according to (Melone et al, 2005). The percentage of GLT-1a in close relationship with excitatory terminals in WT and FHM2 mice was estimated by the number of GLT-1a+ puncta overlapping with VGLUT1+ puncta: the channel corresponding to GLT-1a was examined first and all positive puncta were identified; the channel corresponding to VGLUT1 was viewed next and the presence or absence of overlap was noted (Melone et al, 2009). For GLT-1a/α_2/VGLUT1 analysis in WT mice, before examining the overlap between GLT-1a and VGLUT1, the presence or the absence of colocalization of GLT-1a with α_2 was noted. Finally, size of GLT-1a+ puncta overlaying with VGLUT1 was calculated in all subfields of double-labeled material from all experimental groups (Bozdagi et al, 2000; Bragina et al, 2006). Statistical analysis was performed using GraphPrism v.4.0 (GraphPad Software, La Jolla, CA); a = 0.05.

Equipment, settings and image analysis. For confocal microscopy a TCS SL Leica confocal microscope (Leica Microsystems, Wetzlar, Germany) coupled with a Leica Microsystem Confocal software v.2.61 and equipped with Argon and He-Neon lasers, was used to acquire double-and triple-labeled microscopical fields. Each fluorescent was acquired separately as 512 x 512 pixel images (pixel size of 120 nm) with a planapo x63 objective (numerical aperture 1.4) and pinhole 1.0 Airy unit and with a xy scan mode. Excitation and emission wavelenghts of fluorochromes used were: 488 and 490-540, 543 and 570-630, 633 and 670-800 respectively for green (Alexa 488), red (cy-3 and Alexa

555) and blue (Alexa 647) fluorescents. Original images collected on the surface of stained sections (Melone et al., 2005) were in color scale method (tiff format), 8 bit/channel with an original resolution of 72 dpi (512 x 512 pixels; 18.06 x 18.06 cm).

For each acquired field, a merged image (called composite) was obtained using ImageJ v.1.46r (NIH, USA). Then, randomly selected subfields of 20 × 20 μm were obtained (by ImageJ) from the composite image and, analysis of positive (+) puncta (i.e. GLT-1a, VGLUT1+ puncta in double-labeled material and GLT-1a, α_2, VGLUT1+ puncta in triple-labeled material) was performed (all steps indicate below were performed by ImageJ). The analysis of the selected subfields, required several steps of processing, according to previous studies (Melone et al, 2005). Optimal visualization of punctate staining (good separation between contiguous puncta, along with clear contours for each immunopositive puncta) was achieved by setting a threshold for each color channel to the mean pixel value over the field under study (as previously reported, this is a reliable procedure; indeed with threshold values set anywhere between 0.5 and 2 times the mean pixel brightness had virtually no influence on the extent of overlay between puncta) (Melone et al, 2005). In particular, for studying the size of GLT-1/VGLUT-1 related puncta, threshold was set at 1 times the mean pixel brightness of each color channel and applied to all field for GLT-1a and VGLUT1 and for control and experimental groups. Thresholded images were converted to binary images (binary.tif images), watershed function applied and GLT-1a+ puncta overlapping with VGLUT1+ puncta identified by the observer (using yellow pixels as a sign of overlay). From binary images, size of GLT-1a+ puncta (in pixels) was determined by ImageJ functions (software automatically excluded puncta on the edges of fields and puncta below 5 pixels). Then, size of puncta was converted in μm^2 based on the pixel size of acquisition.

Image processing for final illustrations. For final qualitative high resolution illustrations of double- and triple-labeled fields representative of data obtained, original merged

composite (in color scale method, see above) images were converted into RGB method (tiff format; 8 bit/channel) by ImageJ. Then, using Adobe Photoshop CS5 extended (v.12.1), RGB images were properly dimensioned and converted into high resolution images (600 dpi; tiff format, 8 bit/channel). Selected areas of interest were cropped and levels of each color channel of final images were obtained by using the level function; for each color channel a threshold was applied to all field based on its mean pixel brightness (consistently with the method used to quantify puncta; see above).

Immunogold. For epoxy embedding, dehydrated sections were immersed in propylene oxide, infiltrated with an Epon/Spurr resin mixture, sandwiched between Aclar films, and polymerized at 60°C for 48 h. Chips including layers II/III of SI cortex (at least 2/animal), were cut and sectioned, and ultrathin sections (60–80 nm) were mounted on nickel grids. All rinse and diluent solutions were filtered through a 0.45 μm membrane filter before use. To minimize the effects of procedural variables, post-embedding procedure of grids from WT and KI groups and then from KI saline and KI Cef groups was performed in parallel. Briefly, after treatment with 1% para-phenyenediamine in Tris-buffered saline (0.1 M Tris, pH 7.6, with 0.005% Tergitol N P-10 (TBST)), grids were washed in distilled water, incubated for 15 min in blocking solution (1% bovine serum albumine (BSA) in TBST, pH 7.6) and then transferred in TBST (pH 7.6) solution containing GLT-1a made in rabbit primary antibodies (6 μg/mL). The next day, grids were washed in TBST pH 7.6, incubated for 15 min in blocking solution (1% BSA in TBST pH 8.2), transferred to TBST (pH 8.2; 2 h) containing secondary antibodies conjugated to 12 nm gold particles (1:20; 111-205-144, Jackson), washed, stained with uranyl acetate and Sato’s lead, and examined with a Philips EM 208 and CM10 electron microscope (Eindhoven, The Netherlands) coupled to a MegaView-II high resolution CCD camera (Soft Imaging System, Münster, Germany). Gold

particles were not detected when the primary antiserum was omitted; when normal serum was substituted for the immune serum, parse and scattered particles were observed. Optimal concentration of anti-GLT-1a antibodies was sought by testing several dilutions; the concentration yielding the lowest background labeling and immunopositive elements was used for final studies.

Electron microscopy and data analysis. Microscopic fields (original magnification: 50000–85000X; at least 80-100 fields/animal from 10-15 ultrathin sections/animal) were selected when they included at least one immunolabeled astrocytic profile and/or axon terminal associated with an asymmetric synapse with a clear active zone–postsynaptic density complex (Tyler & Pozzo-Miller, 2001). Background was calculated by estimating labeling density over nuclei (Racz & Weinberg, 2004). For determining the relative density of GLT-1a, gold particles within astrocytic profiles, axon terminals and cell nuclei were counted and areas calculated. Gold particles were considered membrane-associated if they were within 15 nm of its extracellular side, and cytoplasmic if they were > 25 nm from the membrane extracellular side (Melone et al, 2009). Comparison of particle densities (i.e., background, total, cytoplasmic and membrane-associated densities) of immunopositive profiles between WT and FHM2 and between FHM2 saline and cetriaxone groups was then performed using GraphPrism.

Equipment, settings and image analysis. For electron microscopy, Philips EM 208 and CM10 electron microscopes (Eindhoven, The Netherlands) coupled to a MegaView-II high resolution CCD camera with Soft Imaging System (Münster, Germany) and iTEM software v 5.1 (Olympus Soft Imaging Solutions, GmbH) were used to acquire microscopical fields from immunogold processed ultrathin sections. Original collected images were in gray scale method (jpg format), 8 bit/channel with an original resolution of 150 dpi (1376 x 1032 pixels; 23.3 x 17.48 cm) for those of the Philips EM 208, and of 300 dpi (2048 x 2048

pixels; 17.34 x 17.34 cm) for those of CM10. For determining the relative density of GLT-1a gold particles, original images were opened with ImageJ; set scale function was applied and then total, cytoplasmic and membrane areas of profiles were calculate, gold particles within the areas counted and data collected.

Image processing for final illustrations. For final qualitative high resolution illustrations of fields representative of data obtained, original images were properly dimensioned and converted into high resolution images (600 dpi; gray scale method, tiff format, 8 bit/channel) by using Adobe Photoshop CS5 extended (v.12.1). Selected areas of interest were cropped and gray levels of final images, were obtained by using the level function with minimal degree of processing.

Western blotting

Total amount of protein in cortical crude synaptic membranes was determined according to the Bradford method (Bradford, 1976) using the Bio-Rad Protein Assay (Bio-Rad Laboratories, GmbH, Munchen, Germany) and a Beckman DU 530 spectrophotometer (Beckman Coulter, Fullerton, CA; 3–4 measurements/homogenate). A standard curve with 2-10 µg of bovine serum albumin was drawn for each dosing run. Curves of increasing concentration were drawn to define a linear range for densitometric analysis (Bragina et al, 2006). Aliquots of homogenates were subjected to SDS-PAGE (3 µg of total protein [tp] for GLT-1 studies; 7 of [tp] for $\alpha 2$ Na^+,K^+ ATPase studies; and 5 of [tp] for both xCT and Kir4.1 studies). To control potein loading, β-actin and β-tubulin were used as housekeeping proteins (Li & Shen, 2013). Based on the known molecular weight of each protein studied and to avoid possible overlaps between bands, β-actin was used as housekeeping protein for GLT-1a and $\alpha 2$ Na^+,K^+ ATPase studies, whereas β-tubulin for xCT, and Kir4.1 experiments. Separated proteins were electroblotted onto nitrocellulose

filters, which were initially washed in phosphate buffered saline with 0.1% Tween 20 (PBS-T; pH7.4); subsequently, they were exposed first to a blocking buffer solution (5% Bio-Rad non-fat dry milk in PBS-T; 1 h), and incubated (2 h at room temperature and then overnight at 4°C) in a solution of 0.1% BSA in PBS-T containing anti-GLT-1a made in rabbit (0.12 μg/ml) and anti-β-actin made in mouse (1:5000; A5441, Sigma-Aldrich, St Louis, MO), or anti-α_2 Na^+,K^+ ATPase made in rabbit (1:350; Merck-Millipore) and anti-β–actin made in mouse (1:5000) primary antibodies, or anti-xCT made in rabbit (1:250; ab93030, Abcam; Cambrige, UK; raised against a synthetic peptide corresponding to N-terminal residues (between 1-50) of mouse xCT; (Van Liefferinge et al, 2016)) and anti-β-tubulin made in mouse (1:2000; T5293, Sigma-Aldrich) primary antibodies or anti-Kir4.1 (1:700; H00003766-M01, Novus Biological, Abingdon, UK; raised against KCNJ10 (NP_002232, A.A.276-380) partial recombinant protein with GSTtag (DFELVLILSGTVESTSATCQVRTSYLPEEILWGYEFTPAISLSASGKYIADFSLFDQVVKVA SPSGLRDSTVRYGDPEKLKLEESLREQAEKEGSALSVRISNV); (Tong et al, 2014)) and anti-β-tubulin made in mouse (1:2000) primary antibodies. The following day, filters were washed with PBS-T and then exposed to appropriate secondary antibodies (Jackson) dissolved in PBS-T. Bands were visualized by the SuperSignal West Pico chemiluminescent substrate (Bragina et al., 2006). Homogenates from each animal were experimented 4-6 times for each antigen. Intensity data were calculated as ratios of GLT1a/β-actin, α_2 Na^+,K^+ ATPase/β-actin, xCT/β-tubulin, and Kir4.1/β-tubulin (Alhaddad et al, 2014; Melzer et al, 2008). Comparison between saline and cetriaxone measures for each antigen was performed using GraphPrism.

Acquisition and quantification of bands. Immnunoreactive bands were visualized by Bio-Rad Chemidoc and Quantity One software v.4.1. Optimal time of exposure during acquisition, was set for each antigen based on the appearance of few saturated pixel in

immunoreactive bands with a visible gray background. For GLT-1a and $\alpha 2$ Na^{+},K^{+} ATPase studies, optimal exposure time for GLT-1a and $\alpha 2$ Na^{+},K^{+} ATPase corresponded to that of β-actin detection whereas for both xCT and Kir4.1, exposure time was different to that of β-tubulin detection (for this reason illustrative images of western blottings of GLT-1a and $\alpha 2$ Na^{+},K^{+} ATPase and β-actin there is no cropping between bands, whereas for those of xCT and Kir4.1 and β-tubulin there is cropping between bands). Original images of acquisition (1sc format) were used to quantify the intensity of bands by Quantity One tools. Collected values were used to calculate ratios of GLT1a/βactin, $\alpha 2$ Na^{+},K^{+} ATPase/β-actin, xCT/β-tubulin, and Kir4.1/β-tubulin.

Image processing for final illustrations. Original images (1sc format) were exported in tiff format (resolution of 97.5 dpi, gray scale method, 8 bit/channel, 20 x 13.3 cm) by Quantity One software. For final qualitative illustrations of data, original images were properly cropped (without excluding additional bands), dimensioned and converted into high resolution images (600 dpi; gray scale method, tiff format, 8 bit/channel) by using Adobe Photoshop CS5 extended (v.12.1). Gray levels of final images, were obtained by using the level function with minimal degree of processing.

Appendix References

Alhaddad H, Das SC, Sari Y (2014) Effects of ceftriaxone on ethanol intake: a possible role for xCT and GLT-1 isoforms modulation of glutamate levels in P rats. *Psychopharmacology* **231:** 4049-4057

Bozdagi O, Shan W, Tanaka H, Benson DL, Huntley GW (2000) Increasing numbers of synaptic puncta during late-phase LTP: N-cadherin is synthesized, recruited to synaptic sites, and required for potentiation. *Neuron* **28:** 245-259

Bragina L, Melone M, Fattorini G, Torres-Ramos M, Vallejo-Illarramendi A, Matute C, Conti F (2006) GLT-1 down-regulation induced by clozapine in rat frontal cortex is associated with synaptophysin up-regulation. *J Neurochem* **99:** 134-141

CHESLER M (2003) Regulation and Modulation of pH in the Brain. *Physiological Reviews* **83:** 1183-1221

DeSilva TM, Borenstein NS, Volpe JJ, Kinney HC, Rosenberg PA (2012) Expression of EAAT2 in neurons and protoplasmic astrocytes during human cortical development. *J Comp Neurol* **520:** 3912-3932

Dugue GP, Dumoulin A, Triller A, Dieudonne S (2005) Target-dependent use of co-released inhibitory transmitters at central synapses. *J Neurosci* **25:** 6490-6498

Golovina VA, Song H, James PF, Lingrel JB, Blaustein MP (2003) Na+ pump alpha 2-subunit expression modulates Ca2+ signaling. *Am J Physiol Cell Physiol* **284:** C475-486

Ikeda K, Onaka T, Yamakado M, Nakai J, Ishikawa TO, Taketo MM, Kawakami K (2003) Degeneration of the amygdala/piriform cortex and enhanced fear/anxiety behaviors in sodium pump alpha2 subunit (Atp1a2)-deficient mice. *J Neurosci* **23:** 4667-4676

Juhaszova M, Blaustein MP (1997) Na+ pump low and high ouabain affinity alpha subunit isoforms are differently distributed in cells. *Proc Natl Acad Sci U S A* **94:** 1800-1805

Li R, Shen Y (2013) An old method facing a new challenge: Re-visiting housekeeping proteins as internal reference control for neuroscience research. *Life Sciences* **92:** 747-751

Melone M, Bellesi M, Conti F (2009) Synaptic localization of GLT-1a in the rat somatic sensory cortex. *Glia* **57:** 108-117

Melone M, Burette A, Weinberg RJ (2005) Light microscopic identification and immunocytochemical characterization of glutamatergic synapses in brain sections. *J Comp Neurol* **492:** 495-509

Melzer N, Meuth SG, Torres-Salazar D, Bittner S, Zozulya AL, Weidenfeller C, Kotsiari A, Stangel M, Fahlke C, Wiendl H (2008) A ?-Lactam Antibiotic Dampens Excitotoxic Inflammatory CNS Damage in a Mouse Model of Multiple Sclerosis. *PLoS ONE* **3:** e3149

Minelli A, Castaldo P, Gobbi P, Salucci S, Magi S, Amoroso S (2007) Cellular and subcellular localization of Na+–Ca2+ exchanger protein isoforms, NCX1, NCX2, and NCX3 in cerebral cortex and hippocampus of adult rat. *Cell Calcium* **41:** 221-234

Moseley AE, Lieske SP, Wetzel RK, James PF, He S, Shelly DA, Paul RJ, Boivin GP, Witte DP, Ramirez JM, Sweadner KJ, Lingrel JB (2003) The Na,K-ATPase alpha 2 isoform is expressed in neurons, and its absence disrupts neuronal activity in newborn mice. *J Biol Chem* **278:** 5317-5324

Omrani A, Melone M, Bellesi M, Safiulina V, Aida T, Tanaka K, Cherubini E, Conti F (2009) Up-regulation of GLT-1 severely impairs LTD at mossy fibre--CA3 synapses. *J Physiol* **587:** 4575-4588

Racz B, Weinberg RJ (2004) The subcellular organization of cortactin in hippocampus. *J Neurosci* **24:** 10310-10317

Rothstein JD, Martin L, Levey AI, Dykes-Hoberg M, Jin L, Wu D, Nash N, Kuncl RW (1994) Localization of neuronal and glial glutamate transporters. *Neuron* **13:** 713-725

Tong X, Ao Y, Faas GC, Nwaobi SE, Xu J, Haustein MD, Anderson MA, Mody I, Olsen ML, Sofroniew MV, Khakh BS (2014) Astrocyte Kir4.1 ion channel deficits contribute to neuronal dysfunction in Huntington's disease model mice. *Nat Neurosci* **17:** 694-703

Tottene A, Conti R, Fabbro A, Vecchia D, Shapovalova M, Santello M, van den Maagdenberg AMJM, Ferrari MD, Pietrobon D (2009) Enhanced Excitatory Transmission at Cortical Synapses as the Basis for Facilitated Spreading Depression in Ca(v)2.1 Knockin Migraine Mice. *Neuron* **61:** 762-773

Tyler WJ, Pozzo-Miller LD (2001) BDNF enhances quantal neurotransmitter release and increases the number of docked vesicles at the active zones of hippocampal excitatory synapses. *J Neurosci* **21:** 4249-4258

Van Liefferinge J, Bentea E, Demuyser T, Albertini G, Follin-Arbelet V, Holmseth S, Merckx E, Sato H, Aerts JL, Smolders I, Arckens L, Danbolt NC, Massie A (2016) Comparative analysis of antibodies to xCT (Slc7a11): Forewarned is forearmed. *Journal of Comparative Neurology* **524:** 1015-1032
