# Supplementary material for: Defective glutamate and K+ clearance by cortical astrocytes in familial hemiplegic migraine type 2
Source: EMBO Mol Med. 2016 Jun 27;8(8):967–86. doi: 10.15252/emmm.201505944 (PMC4967947; doi:10.15252/emmm.201505944)
Supplement: Supplementary file 2 — Expanded View Figures PDF [file EMMM-8-967-s002.pdf]

Expanded View Figures

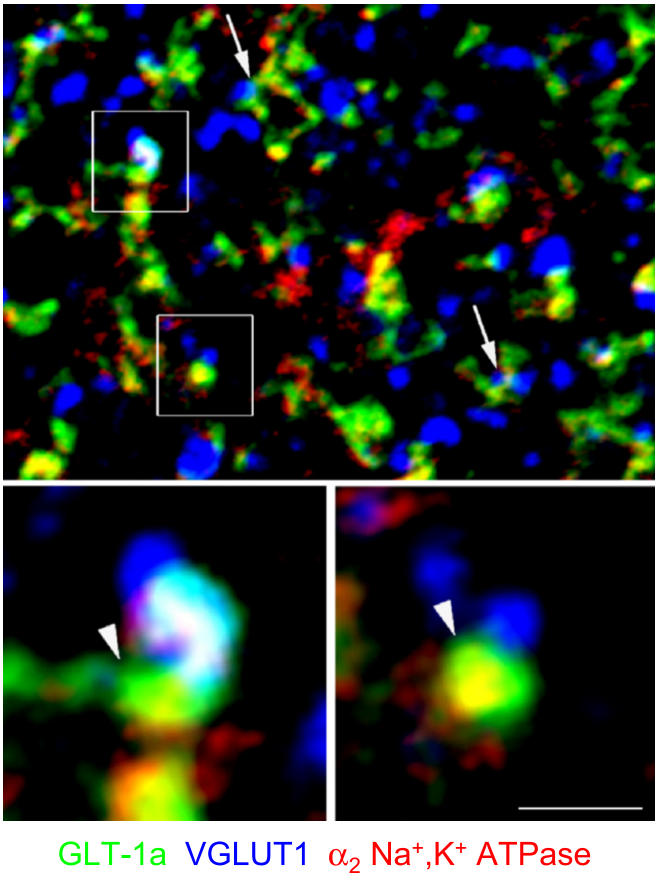

Figure EV1. Colocalization of GLT-1a and α_2 NKA in the vicinity of cortical glutamatergic synapses in WT mice.
Simultaneous visualization of GLT-1a (green), α_2 Na^+,K^+ ATPase (α_2 NKA) (red), and VGLUT1 (blue) immunoreactivity puncta in a section of first somatic sensory cortex (SI) of a WT mouse. Confocal microscopy inspection of fields reveals a high degree of colocalization between GLT-1a and α_2 NKA in GLT-1a$^+$ puncta overlaying with VGLUT1$^+$ puncta (arrows). Framed regions (enlarged below) show examples of overlapping GLT-1a/VGLUT1 puncta that are colocalized with α_2 NKA. Data obtained from 32 fields of 20 × 20 μm from 2 P32 WT mice (3 sections/animal) revealed that 81 ± 2% of overlapping GLT-1a/VGLUT1 puncta colocalized with α_2. Scale bar: 3.5 μm for upper panel and 1 μm for enlarged framed areas. All microscopic fields are from layers II/III.

Source data are available online for this figure.

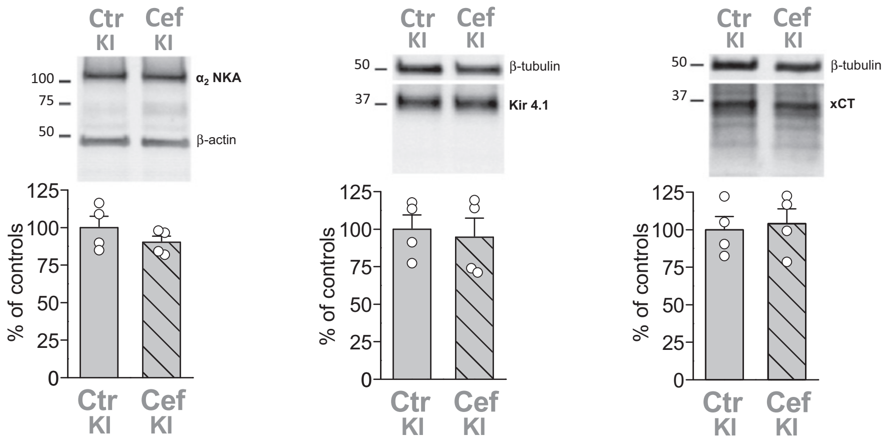

Figure EV2. Ceftriaxone treatment does not modify cortical levels of α_2 NKA, Kir4.1 and xCT in FHM2 KI mice.
Western blotting of α_2 NKA, Kir 4.1, and xCT in cortical crude synaptic membranes of P39 FHM2-KI mice following Cef treatment for 8 days. Protein levels are similar in Cef-treated (Cef KI, $N = 4$) and saline-injected (Ctr KI, $N = 4$); Mann–Whitney U-test: $P = 0.34$, 0.83, and 0.83 for α_2 NKA, Kir 4.1, and xCT, respectively.

Source data are available online for this figure.

© 2016 The Authors
