# Supplementary material for: Defective glutamate and K+ clearance by cortical astrocytes in familial hemiplegic migraine type 2
Source: EMBO Mol Med. 2016 Jun 27;8(8):967–86. doi: 10.15252/emmm.201505944 (PMC4967947; doi:10.15252/emmm.201505944)
Supplement: Supplementary file 3 — Source Data for Expanded View and Appendix [file EMMM-8-967-s012.zip › Source_data_for_Expanded_View_and_Appendix/Source_data_for_Appendix_Figure_S1.pdf]

Appendix Figure S1 Panel B Source Data

Stimulation	STC τ_{decay}	STC amplitude
100	6.19	44
300	6.27	78
600	6.18	117
800	6.28	139
