# Supplementary material for: Defective glutamate and K+ clearance by cortical astrocytes in familial hemiplegic migraine type 2
Source: EMBO Mol Med. 2016 Jun 27;8(8):967–86. doi: 10.15252/emmm.201505944 (PMC4967947; doi:10.15252/emmm.201505944)
Supplement: Supplementary file 3 — Source Data for Expanded View and Appendix [file EMMM-8-967-s012.zip › Source_data_for_Expanded_View_and_Appendix/Source_data_for_Appendix_Figure_S2.pdf]

Appendix Figure S2 Panel A Left Source Data

WT Thresh	KI Thresh
250	150
250	130
250	210
270	190
230	110
230	190
230	150
250	130
250	130
250	150
270	130
250	150
230	110
230	190
210	190
250	130
210	130
270	110
230	110
250	150
	170
	90
	170
	150
	170
	170

Appendix Figure S2 Panel A Right Source Data

WT velocity	KI velocity
3.13	4.44
2.78	4.65
3.83	4.7
3.24	4.54
3.36	4.28
3.24	4.19
3.49	3.91
3.21	4.21
3.33	4.62
3.54	4.1
2.81	4.5
3.23	3.46
3.68	5.03
3.43	4.23
3.58	4.19
3.44	4.43
3.79	3.87
3.8	4.38
3.73	4.06
3.64	4.14
	3.98
	4.45
	4.32
	4.52
	4.28
	4.11

Appendix Figure S2 Panel B Left Source Data

WT Thresh	KI Thresh
210	150
310	210
250	190
270	230
250	190
250	210
290	230
270	210
250	230
270	210
270	210
270	190
290	230
290	210
270	170
	230
	250

Appendix Figure S2 Panel B Right Source Data

WT velocity	KI velocity
2.81	3.35
2.58	3.58
2.67	3.53
2.98	3.64
2.61	3.57
2.78	3.79
2.86	3.69
2.82	3.33
2.67	3.28
2.76	3.33
2.93	2.91
2.79	3.26
3.04	2.92
2.5	3.35
2.73	3.15
	2.96
	3.01
