# Supplementary material for: Defective glutamate and K+ clearance by cortical astrocytes in familial hemiplegic migraine type 2
Source: EMBO Mol Med. 2016 Jun 27;8(8):967–86. doi: 10.15252/emmm.201505944 (PMC4967947; doi:10.15252/emmm.201505944)
Supplement: Supplementary file 3 — Source Data for Expanded View and Appendix [file EMMM-8-967-s012.zip › Source_data_for_Expanded_View_and_Appendix/Source_data_for_Figure_EV1.pdf]

Expanded view Fig 1 (EV1) Source Image

original 72 dpi image (color scale method)

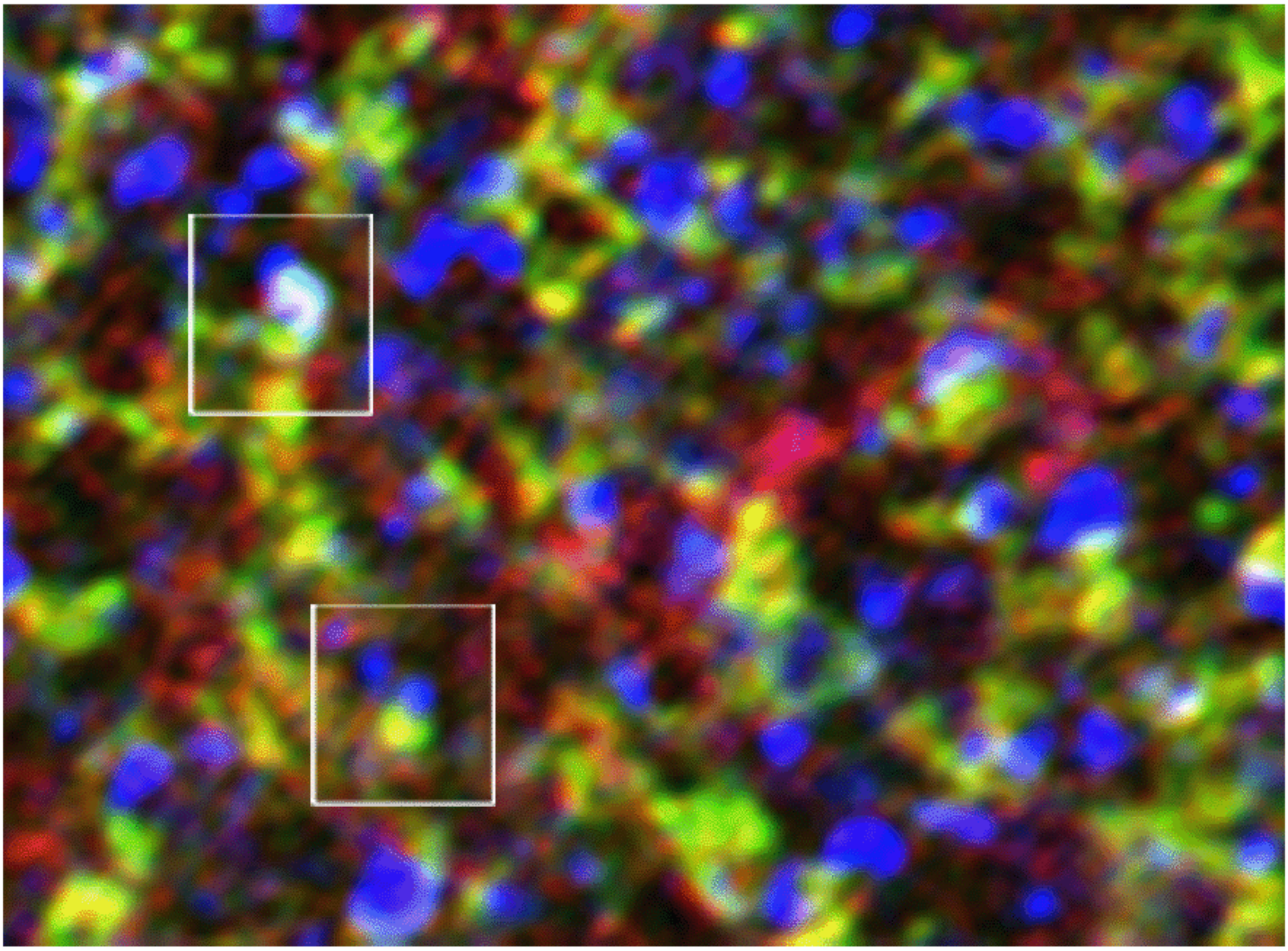

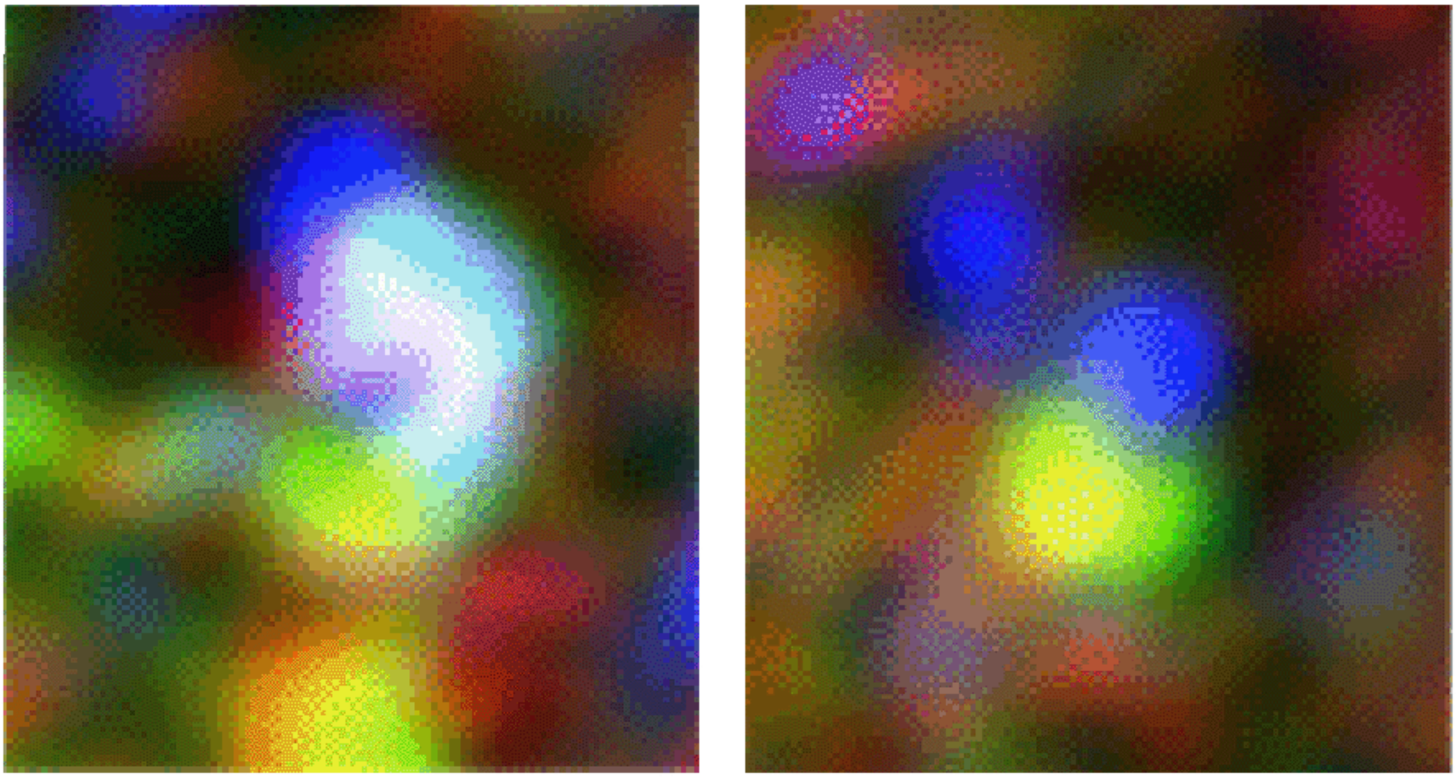

enlarged framed regions
