# Supplementary material for: Defective glutamate and K+ clearance by cortical astrocytes in familial hemiplegic migraine type 2
Source: EMBO Mol Med. 2016 Jun 27;8(8):967–86. doi: 10.15252/emmm.201505944 (PMC4967947; doi:10.15252/emmm.201505944)
Supplement: Supplementary file 3 — Source Data for Expanded View and Appendix [file EMMM-8-967-s012.zip › Source_data_for_Expanded_View_and_Appendix/Source_data_for_Figure_EV2.pdf]

Expanded view Fig 2 (EV2) Upper Panels (left, middle, right) Source Images

Original resolution images (97.5 dpi; generated by Quantity One Chemidoc software)

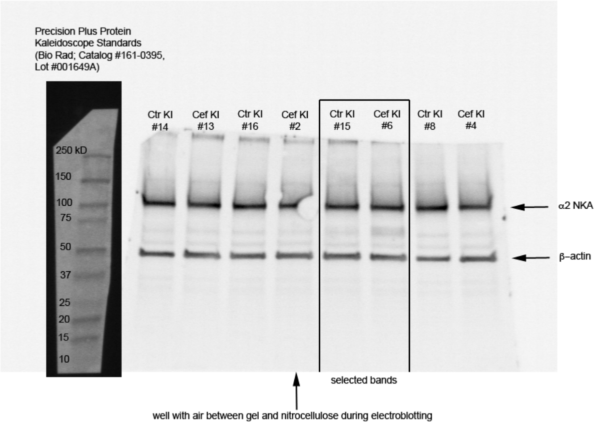

Expanded view Fig 2 (EV2) Lower Left Panel Source Data

Ctr KI	Cef KI
89.79136	96.63639
108.9252	81.8923
84.9056	97.91363
116.3778	84.22358

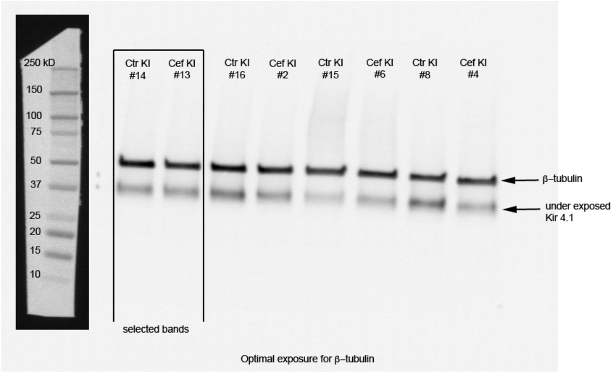

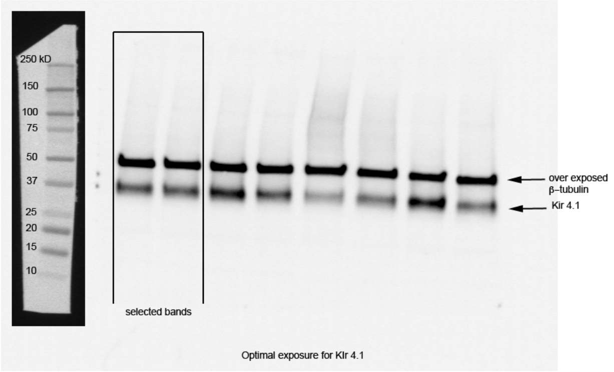

Expanded view Fig 2 (EV2) Lower Middle Panel Source Data

Ctr KI	Cef KI
91.30003	113.9927
117.6977	74.03242
77.40655	119.2855
113.5958	71.18756

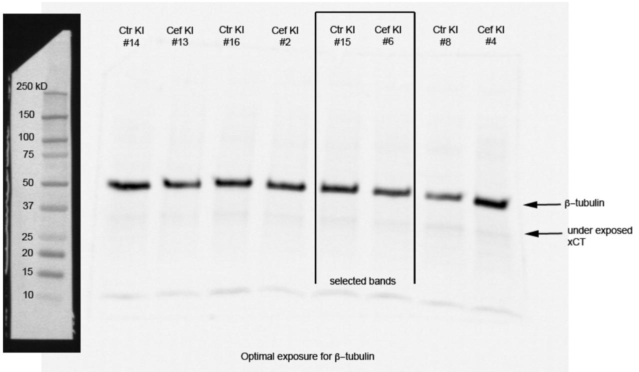

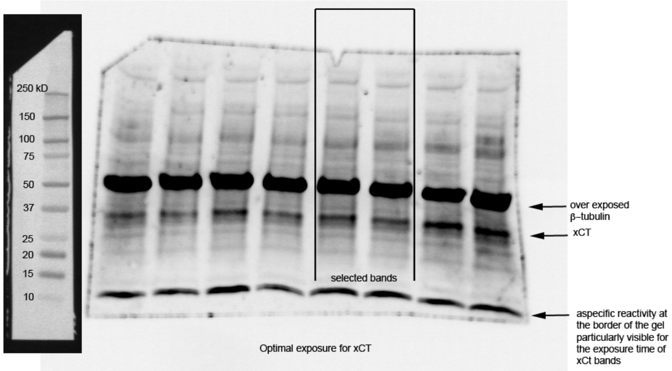

Expanded view Fig 2 (EV2) Lower Right Panel Source Data

Ctr KI	Cef KI
82.4395	122.5169
90.33882	78.52856
105.1307	116.7473
122.091	99.28364

a) After electroblotting column Kaleidoscope Standard was cut and separated from the nitrocellulose and then acquired by Chemidoc in white mode using Epi White light source. The light 10kD band represents the end point of the electrophoresis run.

b) Each Ctr and Cef case was experimented in 4-6 gels (see material and methods for details on data analysis)
