# Supplementary material for: Defective glutamate and K+ clearance by cortical astrocytes in familial hemiplegic migraine type 2
Source: EMBO Mol Med. 2016 Jun 27;8(8):967–86. doi: 10.15252/emmm.201505944 (PMC4967947; doi:10.15252/emmm.201505944)
Supplement: Supplementary file 5 — Source Data for Figure 1 [file EMMM-8-967-s003.pdf]

Figure 1 Panel B Source Data

WT STC τ_{decay}	KI STC τ_{decay}
6.92	7.66
6.55	6.57
7.59	8.91
5.84	9.46
6.36	8.84
6.525	7.24
7.61	7.18
7.11	7.89
6.32	8.01
6.00	
7.46	
6.28	
7.87	

Fig 1 Panel C Source Data

WT STC τ_{decay}	KI STC τ_{decay}
6.21	7.09
6.97	6.67
7.05	8.098
6.39	6.88
4.72	8.97
5.95	8.42
6.49	6.93
7.66	6.95
7.94	8.21
5.76	7.96
6.51	9.2
6.99	6.51
6.28	8.52
5.77	7.06
6.1	8.33
7.28	9.58
7.64	7.57
6.03	8.47
7.3	7.34
5.85	7.55
6.49	8.32
6.16	7.16
6.44	7.29
6.02	7.92
6.19	7.21
6.39	8.16
5.95	8.74
6.44	
