# Supplementary material for: Defective glutamate and K+ clearance by cortical astrocytes in familial hemiplegic migraine type 2
Source: EMBO Mol Med. 2016 Jun 27;8(8):967–86. doi: 10.15252/emmm.201505944 (PMC4967947; doi:10.15252/emmm.201505944)
Supplement: Supplementary file 6 — Source Data for Figure 2 [file EMMM-8-967-s004.pdf]

Figure 2 Panel B Left Source Data

WT τ_{10} 50Hz	KI τ_{10} 50 Hz
7.92	9.66
6.55	8.88
6.43	10.04
8.52	10.28
6.95	9.89
6.55	9.13
6.94	8.86
8.38	9.48
8.15	10.09
7.24	8.23
8.04	9.06
7.08	8.45
8.04	9.78
6.65	10.47
8.09	9.17
8.51	10.38
7.33	10.19
7.45	11.94
7.07	12.62
7.05	8.83
7.09	10.73
9.07	
8.78	

Figure 2 Panel B Right Source Data

WT τ_{10} 100Hz	KI τ_{10} 100Hz
6.67	9.95
6.72	10.19
7.28	10.94
7.75	11.87
8.29	11.43
8.36	12.75
8.91	8.50
8.29	10.61
8.13	10.28
7.57	11.71
8.85	10.63
7.19	14.75
8.20	9.60
10.38	11.87
9.03	
9.25	
7.42	
7.34	
