# Supplementary material for: Defective glutamate and K+ clearance by cortical astrocytes in familial hemiplegic migraine type 2
Source: EMBO Mol Med. 2016 Jun 27;8(8):967–86. doi: 10.15252/emmm.201505944 (PMC4967947; doi:10.15252/emmm.201505944)
Supplement: Supplementary file 7 — Source Data for Figure 3 [file EMMM-8-967-s005.pdf]

Fig 3 Panel A Source Images

original 72 dpi images (color scale method)

WT KI

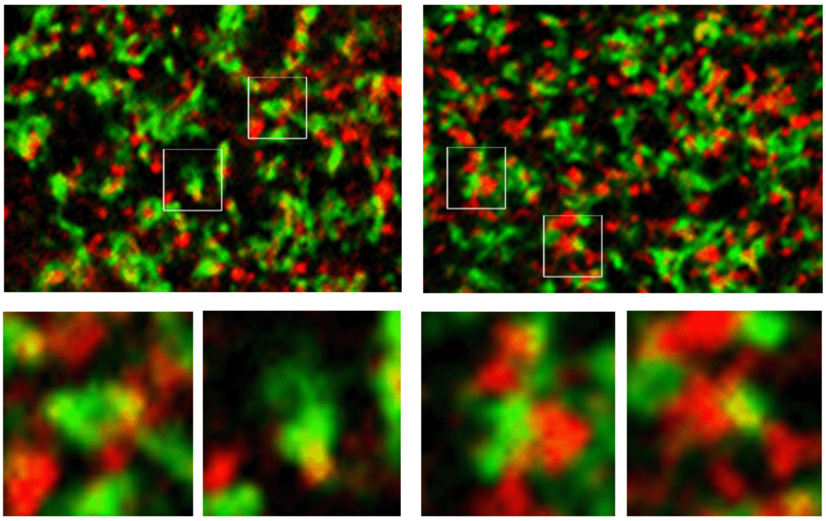

enlarged framed regions

Fig 3 Panel B Left Source Data

WT	KI
48.84	35.9
53.66	63.33
38.46	42.22
52.63	54.05
48.57	51.35
50.00	65.63
64.00	37.93
35.85	61.54
66.67	43.94
34.48	70.73
58.49	40.63
51.72	48.94
33.33	41.82
45.95	36.96
49.43	31.25
36.23	45.16
59.98	38.13
48.79	48.21
	37.34
	43.65
	36.23
	43.55

Fig 3 Panel B Right Source Data

WT			KI		
0.8928	0.432	0.9648	0.144	0.6048	0.4608
0.1152	1.368	0.4464	0.1152	0.5184	0.36
0.8928	0.2448	0.7776	0.5184	0.432	0.072
0.1008	0.36	0.4176	0.8064	0.2304	0.2736
0.1872	0.6048	0.4032	0.288	0.6912	0.2592
0.5472	0.6624	0.4032	1.6128	0.4896	0.8064
0.2448	0.2016	0.5328	0.3312	0.3744	0.1584
0.2592	0.4464	0.1152	0.2016	0.216	0.2016
1.0944	0.072	0.2736	0.1008	0.4608	0.2016
0.8064	0.864	0.8208	0.2736	1.1376	0.144
0.9504	0.0864	0.4752	0.3456	0.288	0.8352
0.36	0.2592	0.7488	0.5616	0.216	0.5472
0.2016	0.1296	0.3312	0.4752	0.1296	0.2592
0.7488	0.2592	0.36	0.5904	0.2304	0.3888
0.5616	0.2016	0.4896	0.432	0.1296	0.2448
0.8064	0.1584	0.0864	0.072	0.8352	0.3888
1.5264	0.8496	0.3744	0.9072	0.3168	0.2016
0.432	1.5552	0.2304	0.2448	0.216	0.648
0.4896	1.1952	0.2016	0.1296	0.2448	0.2304
0.7344	0.432	0.8208	0.3168	0.3456	0.8496
0.6048	1.0656	0.8928	0.2736	0.2448	0.9216
0.2592	0.4032	0.3744	1.3248	0.3456	0.3456
0.3744	0.3888	0.6192	0.36	0.3024	0.4464
0.288	0.5472	0.5184	0.6912	0.2016	0.5904
0.1728	0.1008	0.8928	0.6048	0.4608	0.2016
0.2736	0.6048	0.3312	0.1872	0.2592	1.6272
0.3456	1.7136	0.5328	0.5328	0.3024	0.2592
1.2672	0.5472	0.3456	0.8208	0.4032	0.7488
0.2448	0.1296	0.4032	0.2592	0.1584	0.8928
0.8784	0.4464	1.08	0.5328	0.2592	0.5328
1.2672	0.2736	0.4464	0.3888	0.3456	0.3744
1.008	0.2448	1.2384	0.5184	0.1728	0.3456
0.9792	0.1296	0.5616	0.1584	0.2304	0.7632
0.5184	0.36	0.3744	0.1584	0.2448	0.3744
0.576	0.6624	0.288	0.2592	0.648	0.6336
0.72	0.7776	1.008	0.216	0.8064	0.5184
0.3312	0.3312	0.288	0.1872	0.4032	0.3168
0.144	0.2016	0.3744	0.2448	0.3312	0.9648
0.072	0.3024	0.5184	0.36	0.3456	0.4464
0.4752	0.5184	0.8928	0.6624	0.7056	0.6336
1.296	0.1584	0.4896	0.3024	0.1296	0.4896
0.2448	1.8	0.2304	0.5184	0.6336	0.2304
1.7136	0.4896	0.792	0.1008	1.9728	0.504
0.4896	0.1296	1.008	0.3168	1.6128	0.2304
0.144	0.2592	0.5904	0.504	1.008	0.4464
0.4176	0.2016	0.9792	0.1584	0.1728	0.504
0.4032	1.4112	0.864	0.1296	0.6336	0.3456
0.6048	0.1584	0.3888	0.4896	0.2592	0.5904
0.4032	1.7136	0.5328	0.5184	1.6848	0.5328
0.6048	1.0512	0.3456	0.936	0.648	0.7632
1.08	0.2736	0.936	0.1296	1.0512	0.6048
0.3024	0.3312	0.792	0.6912	0.0864	1.3824
0.1728	0.4608	0.8352	0.9648	0.5616	0.216
0.2448	0.2592	0.6192	0.576	0.3744	
1.4544	1.1232	0.5904	0.2736	0.9936	
0.216	0.1152	0.5472	0.4176	0.2448	
0.1008	0.2016	0.8208	0.3456	0.1008	
0.9936	1.8	0.7488	0.4176	0.3024	
