# Supplementary material for: Defective glutamate and K+ clearance by cortical astrocytes in familial hemiplegic migraine type 2
Source: EMBO Mol Med. 2016 Jun 27;8(8):967–86. doi: 10.15252/emmm.201505944 (PMC4967947; doi:10.15252/emmm.201505944)
Supplement: Supplementary file 8 — Source Data for Figure 4 [file EMMM-8-967-s006.pdf]

Figure 4 Panel A Left Source Images

original 150 dpi images

WT

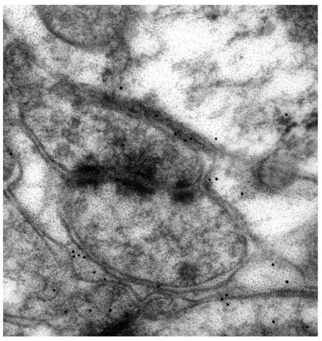

KI

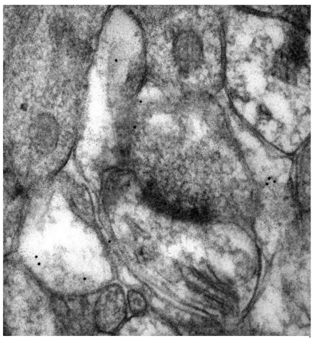

Figure 4 Panel B Left Source Images

WT

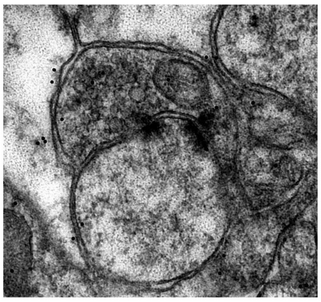

KI

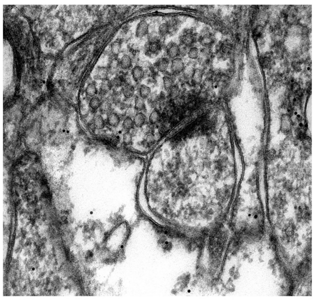

Figure 4 Panel A Right Source Images

WT								
t			cyt			mem		
11.90476	16.66667	35.55556	15.625	0	27.39726	0	45.45454	50.63291
57.14286	8.333333	6.21118	40	0	0	100	30	22.72727
8.695652	10.41667	4.62963	10	0	0	0	40	13.33333
230.7692	12.5	10.20408	0	17.54386	3.095975	500	0	43.47826
13.1579	9.090909	12.37624	0	5.882353	0	121.2121	20	73.52941
6.060606	8.695652	3.537736	0	5.263158	2.574003	32.78688	25	14.08451
15.96806	55.55556	21.2766	6.880734	105.2632	0	76.92308	0	74.07407
3.831418	5.882353	25	4.166667	6.818182	40	0	0	0
8.547009	21.2766	17.69912	6.472492	0	0	23.80952	27.02703	83.33334
90.90909	8.108109	8.086253	153.8462	8.196721	3.076923	0	7.692307	43.47826
29.41176	9.090909	43.47826	0	0	0	187.5	24.39024	52.63158
14.08451	14.28571	4.395605	0	5.714286	5.36193	52.63158	57.14286	0
54.05405	40	1.426533	37.03704	0	1.587302	100	100	0
333.3333	5.882353	100	500	0	0	250	16.66667	153.8462
6.633499	4.545455	6.263048	3.571429	5.263158	2.347418	46.51163	0	37.73585
71.42857	8.235294	25.97403	0	2.985075	23.25581	200	27.77778	29.41176
16.80672	8.695652	2.118644	14.49275	5.555555	0	32.25806	20	17.24138
9.523809	21.42857	272.7273	0	11.49425	0	52.63158	37.73585	300
13.98601	90.90909	27.02703	0	38.46154	0	86.95652	137.931	64.51613
17.85714	16.94915	19.60784	12.19512	23.80952	0	33.33333	0	58.82353
17.04545	61.72839	11.76471	0	70.17544	0	115.3846	41.66667	50
4.62963	17.64706	13.39286	0	14.28571	0	38.46154	33.33333	83.33334
125	28.57143	4.329004	0	55.55556	0	250	0	44.11765
11.53213	40	2.567394	1.886792	37.03704	1.472754	77.92208	43.47826	10
7.874016	7.936508	28.57143	4.62963	0	71.42857	26.31579	38.46154	47.61905
35.29412	20.20202	18.86792	21.2766	0	0	52.63158	47.61905	58.82353
20	3.703704	50	25	0	38.46154	0	25	71.42857
18.18182	58.82353	66.66666	0	0	0	50	83.33334	83.33334
19.60784	8.333333	24.19355	0	0	0	40	41.66667	54.54546
52.63158	68.18182	9.615385	80	52.63158	0	0	80	38.46154
18.60465	27.02703	15.625	11.42857	0	0	50	35.71429	54.05405
33.33333	5.357143	52.63158	0	2.222222	0	75	18.18182	76.92308
10	20	43.47826	0	0	46.51163	33.33333	29.41176	38.46154
10.20408	30.29088	90.90909	14.92537	133.3333	0	0	142.8571	142.8571
44.44444	7.142857	8.982036	33.33333	0	3.448276	66.66666	23.25581	45.45454
2.702703	200	50	3.030303	0	0	0	214.2857	111.1111
19.12568	100	4.56621	19.80198	0	5.747127	15.87302	117.6471	0
22.72727	33.33333	6.369427	0	0	8.196721	66.66666	43.47826	0
11.90476	11.11111	4.464286	17.24138	0	0	0	26.31579	34.48276
50	333.3333	2.255639	0	0	1.666667	90.90909	375	7.692307
13.33333	24.09639	2.040816	10	16.39344	2.380952	20	45.45454	0
20	14.45783	13.63636	14.28571	13.51351	5.555555	33.33333	22.22222	50
18.51852	11.45833	25.31646	0	9.876543	41.66667	30.30303	20	0
76.92308	11.11111	11.23596	0	8	13.51351	83.33334	26.66667	0
11.76471	18.60465	25	0	13.88889	0	40	42.85714	100
33.33333	23.52941	2.941176	0	15.38461	0.833333	71.42857	50	18.75
6.896552	32.35294	3.468208	0	29.62963	0	28.57143	42.85714	36.14458
52.63158	6.818182	20.83333	0	4.201681	0	83.33334	30.76923	33.33333
25.64103	166.6667	31.57895	40	0	15.38461	0	250	66.66666
28.98551	22.22222	1.360544	20	0	1.538462	52.63158	47.61905	0
20	97.56097	15.30612	20	0	13.1579	20	148.1481	22.72727
27.77778	12.5	5.263158	0	8.75	7.142857	66.66666	50	0
13.51351	22.22222	6.22084	16.94915	10.20408	9.259259	0	54.05405	9.708738
23.52941	16.66667	1.785714	0	8.695652	1.173709	333.3333	42.85714	13.63636
10.34483	13.04348	1.602564	0	13.51351	0	75	11.11111	17.69912
28.57143	27.27273	5.780347	21.05263	14.28571	0	44.44444	50	19.23077
13.63636	113.6364	2.65252	6.666667	50	0	28.57143	166.6667	20.40816
20	3.571429	33.8983	14.28571	2.083333	33.33333	33.33333	12.5	34.48276
35.71429	8.339651	3.921569	0	4.237288	2.207505	38.46154	50.35971	17.54386
4.878049	12.09677	6.060606	3.030303	7.371007	3.703704	12.5	33.70787	16.66667
5.797101	60.24096	3.546099	5.172414	75	3.90625	9.090909	46.51163	0
25	50	3.095975	23.07692	40	1.74216	33.33333	66.66666	13.88889
20	50	7.44879	13.69863	40	9.345795	37.03704	66.66666	0
8.333333	54.34783	55.55556	0	12.5	0	40	86.53846	95.2381
20	51.94805	2.989537	22.72727	25	0	0	81.08108	27.39726
52.63158	36.64922	8.333333	0	22.72727	4.878049	83.33334	67.79661	28.57143
4.545455	54.54546	3	0	42.85714	1.785714	12.5	75	20
153.8462	22.98851	0.502639	0	17.54386	0	222.2222	33.33333	6.514658
12.57862	22.47191	2.214839	8.77193	15.50388	0.787712	22.22222	40.81633	23.52941
4	9.398497	10.30928	2.325581	4.338395	0	14.28571	42.25352	37.03704
64.51613	63.49206	1.388889	0	71.42857	0	86.95652	57.14286	14.28571
16.66667	8.783784	1.351351	0	6.060606	0	45.45454	31.25	9.090909
8.333333	11.86441	1.652893	0	6	0	30	44.44444	12.5
10.41667	153.8462	5.681818	0	0	0	40	222.2222	21.73913
12.5	3.508772	3.205128	17.54386	3.167063	1.818182	0	12.34568	13.51351
9.090909	1.785714	33.33333	5.882353	0	0	20	14.28571	50
8.695652	38.46154	1.269841	5.263158	0	0.693481	25	71.42857	7.518797
55.55556	9.722222	3.623188	105.2632	5	1.666667	0	33.33333	16.66667
5.882353	8.281573	8.064516	6.818182	5.714286	12.19512	0	15.03759	0
21.2766	37.03704	1.124859	0	35.71429	0	27.02703	38.46154	11.62791
8.108109	20.76125	37.5	8.196721	12.5523	0	7.692307	60	136.3636
9.090909	11.0664	1.899937	0	5.580357	0.70922	24.39024	61.22449	11.83432
14.28571	12.5	19.60784	5.714286	20	0	57.14286	0	60.60606
40	18.42105	1.058201	0	6.451613	0	100	71.42857	15.38461
5.882353	12.69841	1.966292	0	9.259259	1.219512	16.66667	33.33333	10.71429
4.545455	35.08772	1.818182	5.263158	0	1.960784	0	105.2632	0
8.235294	20	4.938272	2.985075	12	2.816901	27.77778	60	20
8.695652	27.43902	20.40816	5.555555	14.81481	0	20	86.20689	29.41176
21.42857	51.54639	5.769231	11.49425	20	2.173913	37.73585	85.10638	33.33333
90.90909	35.92814	43.47826	38.46154	9.523809	0	137.931	80.64516	55.55556
16.94915	21.42857	12.98701	23.80952	18.18182	0	0	33.33333	32.25806
61.72839	27.77778	18.86792	70.17544	0	0	41.66667	66.66666	37.03704
17.64706	81.63265	3.278688	14.28571	105.2632	1.960784	33.33333	66.66666	10
28.57143	27.39726	1.041667	55.55556	20.61856	1.123595	0	40.81633	0
40	16.97793	6.493506	37.03704	8.350731	0	43.47826	54.54546	13.51351
7.936508	6.861063	4.395605	0	3.853565	2.409639	38.46154	31.25	25
20.20202	16.66667	71.42857	0	25	0	47.61905	0	111.1111
3.703704	13.40483	3.10559	0	9.230769	0	25	41.66667	13.69863
58.82353	2.234637	4.255319	0	2.5	0	83.33334	0	19.60784
8.333333	13.48315	6.666667	0	8.333333	1.960784	41.66667	35.29412	33.33333
68.18182	11.36364	2.141328	52.63158	16.66667	0	80	0	23.25581
27.02703	3.921569	3.301887	0	2.272727	2.030457	35.71429	14.28571	20
5.357143	2.04918	1.47656	2.222222	2.347418	0.410509	18.18182	0	10.98901
20	9.756098	24.63054	0	6.060606	6.25	29.41176	25	93.02325
30.29088	6.363636	55.55556	133.3333	4.081633	83.33334	142.8571	25	41.66667
11.90476	23.12139	3.012048	15.625	16.99717	2.257336	0	36.14458	9.090909
57.14286	5.769231	21.42857	40	3.370786	20.20202	100	20	24.39024
8.695652	5.291005	3.703704	10	0	0	0	20.40816	20
230.7692	18.86792	2.097902	0	0	0.757576	500	34.48276	18.18182
13.1579	6.818182	3.10559	0	6.410256	1.41844	121.2121	10	15
6.060606	4.567814	4.819277	0	3.007519	1.449275	32.78688	26.88172	21.42857
15.96806	9.230769	2.985075	6.880734	7.843137	0	76.92308	14.28571	18.18182
3.831418	6.944445	2.061856	4.166667	1.694915	0	0	30.76923	17.3913
8.547009	51.28205	12.98701	6.472492	22.22222	0	23.80952	90.90909	55.55556
90.90909	25.92593	1.030928	153.8462	23.80952	0	0	33.33333	14.28571
29.41176	7.142857	5.555555	0	5.555555	0	187.5	16.66667	40
14.08451	1.287554	24	0	0	0	52.63158	23.07692	62.5
54.05405	17.3913	27.77778	37.03704	12.19512	0	100	30.30303	58.82353
333.3333	52.63158	22.22222	500	43.47826	41.66667	250	66.66666	0
6.633499	17.33102	37.03704	3.571429	15.87302	0	46.51163	22.05882	83.33334
71.42857	10.20408	1.190476	0	0	0	200	22.72727	14.28571
16.80672	10.13513	11.97605	14.49275	3.952569	7.936508	32.25806	46.51163	24.39024
9.523809	9.411765	3.191489	0	6.849315	1.204819	52.63158	25	18.18182
13.98601	7.317073	10	0	5.714286	0	86.95652	16.66667	37.5
17.85714	10.71429	16.66667	12.19512	10	0	33.33333	12.5	33.33333
17.04545	5.172414	6.134969	0	2.12766	0	115.3846	18.18182	31.25
4.62963	2.380952	21.58273	0	0	0	38.46154	14.28571	50.84746
125	2.898551	6.944445	0	1.639344	2.86533	250	12.5	24.09639
11.53213	7.978724	13.33333	1.886792	2.030457	0	77.92208	48.95105	66.66666
7.874016	6.858711		4.62963	1.498127		26.31579	65.04065	
35.29412	15.0289		21.2766	11.87335		52.63158	37.38318	
20	20.97902		25	17.46725		0	35.08772	
18.18182	5.194805		0	3.521127		50	25	
19.60784	8		0	4.08998		40	22.05882	
52.63158	14.28571		80	9.523809		0	28.57143	
18.60465	7.889546		11.42857	2.702703		50	21.89781	
33.33333	6.451613		0	4.347826		75	12.5	
10	16.27907		0	11.76471		33.33333	33.33333	
10.20408	10.98901		14.92537	0		0	26.66667	
44.44444	9.237875		33.33333	6.060606		66.66666	19.41747	
2.702703	16.66667		3.030303	15.38461		0	20	
19.12568	3.594536		19.80198	0.914077		15.87302	13.46801	
22.72727	10.16949		0	4.065041		66.66666	40.81633	
11.90476	10.75269		17.24138	5.194805		0	37.5	
50	31.25		0	15.38461		90.90909	100	
13.33333	47.61905		10	38.46154		20	62.5	
20	22.22222		14.28571	14.28571		33.33333	50	
18.51852	14.96599		0	11.11111		30.30303	38.09524	
76.92308	27.27273		0	0		83.33334	62.5	
11.76471	22.22222		0	9.523809		40	66.66666	
33.33333	9.589041		0	6.349206		71.42857	30	
6.896552	100		0	200		28.57143	66.66666	
52.63158	57.55396		0	40		83.33334	78.125	
25.64103	4.761905		40	2.816901		0	15.38461	
28.98551	20		20	0		52.63158	43.47826	
20	0		20	0		20	0	
27.77778	48.3871		0	54.6875		66.66666	34.48276	
13.51351	27.02703		16.94915	0		0	66.66666	
23.52941	25		0	0		333.3333	39.21569	
10.34483	62.5		0	0		75	130.4348	
28.57143	13		21.05263	6.976744		44.44444	50	
13.63636	20.83333		6.666667	15		28.57143	50	
20	38.88889		14.28571	20		33.33333	133.3333	
35.71429	37.5		0	0		38.46154	75	
4.878049	66.66666		3.030303	100		12.5	50	
5.797101	40		5.172414	29.41176		9.090909	62.5	
25	11.76471		23.07692	10.71429		33.33333	16.66667	
20	25		13.69863	20		37.03704	27.77778	
8.333333	2.5		0	0		40	16.66667	
20	50		22.72727	50		0	50	
52.63158	11.11111		0	0		83.33334	23.80952	
4.545455	30		0	28.57143		12.5	33.33333	
153.8462	29.04564		0	17.75148		222.2222	55.55556	
12.57862	25.71428		8.77193	21.42857		22.22222	42.85714	
4	14.70588		2.325581	0		14.28571	41.66667	
64.51613	38.46154		0	0		86.95652	52.63158	

KI								
t			cyt			mem		
7.142857	21.2766	6.097561	0	0	3.663004	23.25581	55.55556	18.18182
200	71.42857	9.615385	0	0	16.66667	214.2857	125	0
100	5.555555	5.736138	0	6.382979	4.444445	117.6471	0	13.69863
33.33333	1.492537	18.51852	0	1.612903	0	43.47826	0	86.95652
11.11111	47.61905	83.33334	0	0	200	26.31579	111.1111	0
333.3333	12.34568	12.98701	0	0	19.23077	375	47.61905	0
8.810573	8.333333	62.5	0	11.11111	142.8571	51.28205	0	0
9.523809	9.523809	6.557377	0	0	4.065041	37.03704	40	16.94915
12.98701	16.66667	16	0	24.39024	11.11111	50	0	28.57143
1.663893	3.703704	7.407407	0	4.761905	4.761905	20.40816	0	16.66667
22.47191	16.66667	25	0	7.692307	18.18182	68.96552	40	40
4.854369	15.38461	10	3.521127	10	0	20	33.33333	30
6.756757	13.51351	21.62162	4.6875	0	13.98601	20	30.30303	47.61905
6.521739	22.22222	4.081633	4.761905	47.61905	5	25	0	0
29.23977	4.545455	21.73913	9.174312	0	40	64.51613	25	0
16.52892	73.17073	7.407407	0	50	4.166667	55.55556	95.2381	33.33333
27.02703	16.66667	14.38849	43.47826	8.333333	21.2766	0	33.33333	0
21.21212	40	4.854369	25.92593	29.41176	5.649717	0	62.5	0
4.896627	12.5	10.10101	4.618937	16.66667	4.484305	9.433962	0	27.02703
11.11111	8.333333	20.61856	6.369427	0	27.39726	43.47826	25	0
7.142857	6.622517	3.333333	7.936508	0	4.166667	0	17.85714	0
4.081633	11.76471	4.504505	4.444445	14.28571	5.524862	0	0	0
3.947368	230.7692	43.47826	0	0	66.66666	42.85714	272.7273	0
10.34483	3.712871	14.70588	4	3.067485	11.53846	50	6.410256	25
44.44444	16.94915	25.64103	0	0	41.66667	105.2632	47.61905	0
6.486486	15.87302	12.73885	2.466091	0	0	35.08772	45.45454	37.73585
1.694915	14.28571	11.76471	0	0	0	15.38461	42.55319	33.33333
9.680542	6.666667	45.45454	6.928406	0	27.77778	23.9521	33.33333	78.94736
4	37.03704	16.66667	0	60.60606	33.33333	25	0	0
15.87302	12.5	7.317073	0	5	2.941176	47.61905	50	28.57143
6.557377	13.33333	10.71429	4.62963	8.333333	4.761905	11.23596	33.33333	28.57143
8	8.333333	9.090909	0	0	0	10	16.66667	33.33333
2.857143	8.510638	7.407407	3.225806	4.494382	10.30928	0	80	0
3.717472	10	11.11111	0	0	16.66667	14.28571	33.33333	0
7.5	25	9.090909	5.714286	0	0	20	50	25
44.94382	54.54546	3.937008	53.57143	44.44444	5	60.60606	100	0
18.75	8.849558	2.702703	23.07692	11.97605	3.225806	0	0	0
9.090909	14.92537	3.225806	5.172414	0	0	37.5	68.96552	20
23.80952	17.85714	11.14206	10.30928	0	6.756757	68.96552	50	31.74603
33.33333	12.65823	10.86957	28.98551	18.18182	7.8125	47.61905	0	17.85714
9.756098	4.608295	8.928572	2.777778	2.522068	5.494505	60	26.66667	23.80952
26.66667	16.94915	4.201681	25	41.66667	0	33.33333	0	18.51852
6.17284	20.94241	16.66667	0	26.49007	0	46.15385	0	41.66667
15	2.635046	5.681818	14.28571	1.547988	6.688963	20	8.849558	18.86792
3.571429	3.2	12.39669	4.347826	1.934236	0	0	9.259259	58.82353
9.67742	4.376368	4.711425	3.846154	1.259446	4.016064	40	25	9.803922
15.38461	5.128205	14.28571	13.63636	0	0	25	25	45.45454
10.86957	8.571428	12.65823	11.14206	8.333333	0	9.90099	10	37.03704
6.25	22.05882	6.849315	3.846154	29.12621	2.816901	16.66667	0	24.09639
15	4.854369	7.042253	5.882353	3.759398	5.172414	66.66666	11.62791	15.38461
10.83033	25	9.523809	4.444445	0	0	38.46154	33.33333	33.33333
5.714286	12.5	20	0	0	34.48276	40	33.33333	0
13.46154	2.717391	20	4.347826	0	50	83.33334	16.39344	0
19.23077	8.333333	3.191489	13.63636	14.28571	2.409639	50	0	9.090909
9.090909	2.702703	25	7.142857	3.225806	2.564103	20	0	0
15.90909	2.857143	6.451613	11.42857	1.801802	6.25	33.33333	13.04348	7.142857
13.33333	2.283105	14.28571	0	1.515152	0	50	9.523809	57.14286
23.62205	0.793651	4.854369	27.02703	0	0	11.76471	7.142857	27.77778
12.62626	10.75269	10.79137	6.23053	0	9.569378	40	18.86792	14.49275
10	4.166667	4.310345	4.347826	5.882353	2.415459	28.57143	0	20
13.33333	40	6.25	8.333333	66.66666	0	33.33333	0	25.64103
10.52632	2.325581	3.636364	6.25	2.777778	2.083333	33.33333	0	14.28571
16.66667	11.11111	10.69519	10	9.523809	14.08451	50	16.66667	0
27.27273	2.693603	29.41176	14.08451	2.545455	66.66666	51.28205	4.545455	0
25	31.25	25	33.33333	20.40816	41.66667	0	66.66666	0
17.24138	12.5	3.703704	0	0	2.739726	30.30303	18.18182	12.5
26.31579	4.347826	2.5	0	0	2.857143	62.5	25	0
2.980626	2.816901	11.11111	3.478261	0	7.017544	0	25	26.66667
6.666667	4.255319	5	0	2.564103	6.25	33.33333	12.5	0
20.13423	7.142857	7.434944	0	6.521739	8.928572	46.875	10	0
13.33333	6.666667	4.477612	18.51852	4.166667	1.694915	0	16.66667	25
8.130081	8.695652	33.33333	13.1579	0	25.64103	0	40	47.61905
8.333333	29.41176	23.25581	0	0	55.55556	50	50	0
17.85714	2.439024	14.28571	0	0	20	66.66666	11.11111	0
18.18182	1.886792	4.149378	13.33333	1.052632	5.555555	28.57143	9.090909	0
40	2.013423	2.510986	71.42857	1.824818	1.398601	0	4.166667	12.26994
15	12.2449	27.77778	6.060606	10	14.81481	57.14286	22.22222	66.66666
13.95349	7.334963	27.02703	12.98701	2.762431	0	16.39344	42.55319	62.5
8.510638	5.128205	45.45454	7.317073	0	45.45454	16.66667	12.5	45.45454
27.77778	7.692307	6.976744	0	0	5.555555	66.66666	25	14.28571
10	5.555555	102.0408	0	3.333333	133.3333	33.33333	16.66667	88.23529
15.38461	17.44186	50	11.11111	23.07692	0	25	0	100
14.28571	3.773585	54.05405	9.090909	5.882353	222.2222	33.33333	0	0
11.53846	4.081633	4.83871	9.090909	2.777778	3.636364	25	7.692307	14.28571
7.692307	33.33333	15.78947	9.523809	0	6.451613	0	50	57.14286
5.617978	14.86989	9.132421	9.009009	17.93722	13.7931	0	0	0
26.31579	28.57143	25.80645	40	33.33333	24.69136	0	25	27.02703
25	11.25704	12.5	15	11.36364	0	75	10.75269	33.33333
7.692307	12.5	19.80198	0	0	14.28571	33.33333	33.33333	32.25806
10.58201	12.42236	16.66667	7.936508	7.751938	0	15.87302	31.25	33.33333
5.235602	1.030928	4.237288	0	16.66667	0	20.40816	0	25
3.448276	11.11111	5.825243	0	0	2.197802	25	25	33.33333
6.825939	40	28.57143	8.695652	0	0	15.87302	55.55556	66.66666
2.398082	3.125	12.65823	0	1.219512	4.938272	15.87302	14.28571	57.97102
11.49425	1.538462	7.352941	14.42308	0.854701	0	0	7.692307	31.25
12.34568	10.34483	32.25806	0	8.333333	0	41.66667	20	76.92308
12.90323	11.11111	7.194244	15.38461	9.523809	0	0	16.66667	31.57895
2.5	36.36364	3.184713	2.542373	25	3.952569	0	66.66666	0
9.090909	15.38461	15.38461	13.04348	10	29.41176	0	33.33333	0
11.76471	20	7.092198	0	0	0	24.69136	50	22.72727
5.089058	20	3.448276	3.448276	0	0	9.708738	100	16.66667
8.163265	5.555555	0	0	6.451613	0	83.33334	0	0
22.98851	10.90909	11.31222	19.23077	9.302325	9.966778	28.57143	16.66667	14.1844
13.51351	7.407407	3.636364	22.22222	5	0	0	14.28571	18.51852
19.23077	22.22222	0	32.25806	16.66667	0	0	33.33333	0
28.57143	7.98722	2.73224	26.66667	5.524862	1.572327	33.33333	24.09639	10.41667
8.752735	10.56338	6.19195	5.012531	8.080808	3.846154	34.48276	27.39726	15.87302
15.87302	27.52294	1.597444	0	18.18182	0	25.64103	37.03704	9.708738
10	7.058824	7.648183	8.928572	2.857143	1.815981	11.36364	26.66667	-0.88574
36.69725	22.98851	4.255319	13.88889	20	5.319149	81.08108	32.78688	0
21.50538	10.15228	21.2766	16.39344	12.98701	10.41667	31.25	0	44.44444
23.4375	1.43575	6.309148	13.33333	1.179709	0	37.73585	4.115226	29.85075
9.523809	6.861063	4.750594	6.993007	3.976143	5.970149	14.92537	25	0
10.10101	13.33333	2.890173	15.38461	20.40816	1.282051	0	0	17.64706
68.96552	8.247422	4.310345	111.1111	2.415459	5.208333	50	42.25352	0
7.792208	58.82353	19.04762	3.134796	37.03704	18.51852	30.30303	83.33334	19.60784
20.28986	2.034588	5.940594	10.67616	2.412545	3.529412	62.5	0	18.75
32	1.904762	4.024145	22.72727	2.12766	1.176471	54.05405	0	20.83333
7.633588	9.202454	11.18568	5.376344	4.184101	8.108109	13.1579	22.98851	25.97403
2.590674	8.559919	3.030303	3.424658	6.722689	3.389831	0	24.87562	0
2.923977	6.369427	12.82051	3.623188	5.141388	6.451613	0	12.19512	37.5
3.164557	7.434944	5.988024	0	4.484305	8.333333	12.65823	21.73913	0
16.26016	3.821656	4.545455	0	3.003003	0	47.61905	8.403361	22.22222
21.09705	3.937008	4.694836	32.52032	3.418803	2.695418	8.77193	10	18.18182
23.4375	1.818182	4.709576	11.76471	2.040816	3.590664	46.51163	0	12.5
0	9.345795	4.115226	0	0	4.672897	0	36.36364	0
15.38461	5.970149	7.782101	21.73913	0	9.433962	0	24.39024	0
0	13.7931	11.67315	0	17.3913	9.433962	0	0	22.22222
4.792332	22.72727	5.405406	2.34192	24.39024	4.149378	10.05025	18.86792	13.69863
15.30612	7.858546	19.04762	0	4.750594	26.31579	60	22.72727	0
3.333333	6.666667	16.26016	0	10	11.49425	8.264462	0	27.77778
3.021148	7.142857	7.604563	0	0	4.524887	40	57.14286	23.80952
18.51852	14.08451	15.26718	0	7.042253	10.20408	38.46154	23.80952	30.30303
10.36269	3.968254	20.68966	6.896552	0	19.60784	20.83333	16.66667	23.25581
12.65823	5.333333	8.810573	5.154639	4	8.620689	46.51163	10	9.90099
3.018868	7	5.172414	0.833333	2	3.861004	24	50	6.23053
11.36364	8.333333	10	9.302325	2.777778	12.5	20.40816	25	0
26.66667	4.878049	5.194805	0	2.439024	1.5625	45.45454	16.66667	23.07692
1.36612	22.90076	2.01005	1.533742	15.26718	1.639344	0	19.60784	6.25
11.81102	1.025641	21.2766	0	0.769231	33.33333	33.70787	5	0
111.1111	8.333333	16.66667	200	6.666667	29.41176	76.92308	8.333333	0
2.10084	2.727273	4.065041	2.43309	0.909091	3.04414	138.4615	20	12.34568
0.863558	11.11111	33.33333	1.924928	7.407407	0	8.403361	16.66667	50
1.779359	6	10.67616	1.967213	4	4.484305	0	13.33333	34.48276
3.030303	32.96703	13.51351	3.584229	32.96703	16.12903	0	0	0
1.193317	1.754386	6.25	1.432665	0	7.692307	0	12.5	0
6.993007	1.388889	18.18182	0	0	0	22.22222	14.28571	50
7.092198	14.28571	5.747127	9.174312	14.28571	5.263158	0	0	9.090909
3.960396	22.22222	64.51613	2.398082	11.11111	66.66666	11.36364	25	62.5
0.388199	14.28571	7.407407	0.434405	14.28571	4.761905	0	0	16.66667
5.970149	10	6.849315	7.168459	8	10	0	10	0
6.578948	14.28571	12.5	7.692307	14.28571	8.510638	0	0	22.22222
8.474576	105.2632	3.571429	4.938272	52.63158	3.448276	29.85075	90.90909	3.703704
2.105263	9.302325	5.586592	1.207729	4.651163	3.658537	8.196721	20	26.66667
5.154639	14.81481	9.259259	6.25	7.407407	8.888889	0	40	11.11111
1.362398	5.660378	8.888889	0	3.773585	8.571428	10.20408	10	10
4.926108	8	13.69863	5.91716	0	18.34862	0	22.72727	0
13.88889	8.746356	5.076142	0	5.830904	6.289308	27.77778	12.82051	0
4.662004	2.116402	6.666667	2.793296	1.058201	9.090909	14.08451	14.28571	0
2.272727	1.244813	1.923077	0	0.829876	1.438849	2.421308	9.090909	5.882353
14.70588	10.44776	6.25	30.30303	7.462687	5.128205	0	22.22222	11.11111
20.83333	3.043478	9.569378	0	2.173913	12.19512	45.45454	10	0
38.46154	8.333333	44.44444	0	0	0	111.1111	40	83.33334
38.46154	9.090909	8.37521	100	3.030303	4.081633	0	25	28.03738
100	7.692307	1.948052	250	7.692307	1.408451	0	0	8.333333
20	36.36364	6.896552	16.66667	18.18182	0	25	40	21.2766
47.61905	44.77612	3.296703	0	78.94736	2.531646	76.92308	0	8.333333
4.761905	21.73913	16.66667	2.932551	22.72727	12.5	12.65823	20	25
33.33333	2.683363	3.412969	0	32.6087	4.201681	71.42857	0	0
25.64103	20.72539	22.72727	52.63158	21.73913	35.71429	0	18.18182	0
6.666667	11.53846	42.85714	9.090909	5.076142	25.64103	0	31.74603	64.51613
11.49425	28.57143	35.29412	0	42.55319	36.03604	19.60784	0	36.8983
13.88889	7.142857		0	4		37.03704	33.33333	
7.692307	11.11111		4.545455	0		25	20	
14.28571	14.28571		20	0		0	66.66666	
8	33.33333		6.153846	0		20	71.42857	

Figure 4 Panel B Right Source Images

WT								
t			cyt			mem		
37.5	16.80672	4.514673	28.57143	10.98901	5.333333	100	35.71429	0
12.82051	11.62791	6.71141	15.87302	0	5.405406	0	44.44444	12.98701
2.821671	5.830904	1.128668	4.145937	4.983389	0	0	11.90476	13.1579
1.876173	7.662835	4.343105	0	0	2.375297	19.23077	42.55319	25.31646
0.98912	3.846154	8.77193	0	0	9.803922	17.85714	25	16.66667
3.278688	11.62791	2.390438	0	10	2.531646	30.30303	33.33333	0
1.876173	7.894737	3.174603	0	8.823529	2.586207	22.72727	0	10
7.042253	7.692307	7.050529	0	5.714286	5.249344	31.25	25	22.47191
6.060606	20	2.380952	3.571429	25	1.333333	20	0	11.11111
3.125	3.125	1.976285	3.448276	3.703704	2.28833	0	0	0
3.030303	2.857143	3.389831	3.448276	0	0	0	20.83333	6.25
1.754386	7.692307	13.63636	1.923077	9.090909	11.11111	0	0	25
3.703704	4.761905	7.130125	2.702703	0	6.072875	14.28571	33.33333	14.92537
4	62.5	4.132231	4.444445	57.14286	3.636364	0	100	9.090909
5.555555	5.555555	4.398827	3.030303	6.25	2.44898	33.33333	0	21.58273
2.597403	5.882353	15.68627	1.388889	7.142857	9.433962	20	0	46.51163
5	11.49425	3.157895	5.882353	14.28571	2.325581	0	0	11.11111
3.125	6.849315	10	0	0	6.818182	25	30.30303	33.33333
2.777778	9.090909	2.816901	3.225806	5.263158	1.5625	0	33.33333	14.28571
2.564103	3.448276	3.703704	0	1.960784	2.083333	12.5	14.28571	16.66667
2.352941	4.545455	4.651163	1.265823	0	2.702703	16.66667	50	16.66667
4.166667	12.5	4.615385	5	0	3.448276	0	40	14.28571
6.976744	6.666667	6.896552	2.631579	8.333333	4.545455	40	0	30.76923
5.172414	3.333333	3.571429	1.886792	3.703704	1.315789	40	0	25
1.714286	11.11111	5.813953	0.598802	0	5.128205	25	133.3333	12.5
12.5	3.571429	6	0	4.166667	4.347826	66.66666	0	25
8.823529	6.666667	3.157895	10	8	1.190476	0	0	18.18182
3.125	1.851852	5.420054	3.389831	0	6.451613	0	20	0
8.823529	12.5	6.060606	3.225806	14.28571	3.571429	66.66666	0	20
3.278688	6.593407	9.67742	3.571429	4.878049	4	0	22.22222	33.33333
2.564103	13.69863	18.77934	0	9.90099	11.69591	25	32.25806	47.61905
10	15.09434	22.14022	7.407407	12.46537	18.18182	33.33333	41.09589	39.21569
1.785714	14.81481	6.71141	1.923077	8.695652	0	0	50	37.03704
0.862069	5.641026	13.24503	0.909091	4.444445	16.94915	0	13.33333	0
1.851852	12.84109	9.55414	0	7.233273	3.773585	7.692307	57.14286	40.81633
1.149425	4.340278	6.006006	0	1	0	16.66667	0	42.55319
0.725163	12.9199	0.634317	0.76864	9.009009	0.672269	0	37.03704	0
2	20.72539	0	2.320186	13.1579	0	0	48.78049	0
10.98901	11.02941	3.021148	0	8.733624	3.460208	43.47826	23.25581	11.90476
3.095975	8.823529	4.166667	0	8.333333	3.115265	18.86792	12.5	12.82051
8.298756	3.947368	1.104972	10.25641	2.898551	0.598802	0	14.28571	7.142857
5.208333	3.846154	3.278688	0	4.444445	0	28.57143	0	28.57143
0	1.6	0.398883	0	1.424501	0.425532	0	4.166667	0
9.132421	9.615385	0.527983	11.62791	0	0.56338	0	31.25	0
51.28205	6.122449	2.229654	44.94382	4.651163	1.236094	71.42857	16.66667	11.36364
1.54321	13.88889	5.454545	1.745201	10	6.122449	0	33.33333	0
3.826531	2.824859		4.166667	3.067485		0	0	
2.267574	7.042253		0	0		17.24138	33.33333	
0.948767	15.06849		0	14.0625		10.75269	22.22222	
3.134796	5.050505		0	3.370786		19.60784	20	
9.009009	8.247422		0	5.681818		62.5	33.33333	

KI								
t			cyt			mem		
3.669725	5.747127	9.756098	4.210526	6.756757	0	0	0	58.82353
1.040583	9.345795	10	1.172333	12.04819	6.25	0	0	25
13.11475	11.11111	1.428571	7.604563	13.69863	0	47.61905	0	20
12.2449	3.076923	22.22222	9.433962	3.597122	28.57143	30.30303	0	0
6.548176	9.661836	9.090909	5.030181	5.91716	5.555555	26.66667	26.31579	25
1.898734	1.552795	14.28571	2	1.74216	9.090909	0	0	33.33333
23.80952	0.816994	0	22.22222	0	0	33.33333	11.23596	0
1.25	1.01833	6.134969	1.333333	1.135074	7.29927	0	0	0
17.3913	1.430615	10.52632	15	1.592357	7.142857	33.33333	0	20
10.71429	2.504696	5.797101	12.5	2.695418	3.521127	0	0	16.39344
7.692307	2.34192	9.67742	0	0	8	50	18.86792	16.66667
10.34483	3.937008	4.464286	7.594937	0	5.405406	37.5	23.80952	0
11.11111	4.535147	12.73885	8.333333	5.089058	8.064516	33.33333	0	30.30303
5.405406	6.349206	8.130081	6.060606	7.142857	0	0	0	34.48276
8.333333	0.60423	3.629764	0	0	2.173913	66.66666	10.75269	10.98901
6.666667	1.322751	4	7.692307	1.48368	3.061224	0	0	50
0	0.202881	1.875	0	0	0.675676	0	5.235602	16.66667
6.666667	5.30504	7.70416	2.5	6.21118	5.357143	40	0	22.47191
7.142857	6.269592	8.108109	8.333333	3.663004	3.448276	0	21.73913	25
2.325581	2.272727		0	0		25	25	
7.792208	5.333333		7.142857	4.347826		14.28571	16.66667	
1.626016	11.11111		0	13.33333		25	0	
5.714286	3.333333		6.451613	0		0	25	
6.666667	7.142857		2.631579	4		28.57143	33.33333	
10	5		11.76471	0		0	33.33333	
6.666667	4.761905		0	0		33.33333	33.33333	
5.555555	1.162791		0	1.265823		33.33333	0	
5	7.407407		5.555555	0		0	50	
2.857143	3.448276		0	0		25	33.33333	
9.230769	1.754386		7.067138	1.960784		23.80952	0	
5	4.347826		0	5		33.33333	0	
10.6383	2.564103		7.5	2.941176		28.57143	0	
2.12766	4.504505		0	5.263158		25	0	
9.090909	1.098901		5.555555	1.203369		25	0	
8.571428	0.935454		3.225806	1.013171		50	0	
0	2.590674		0	0		0	29.12621	
5.555555	1.618123		0	1.821494		33.33333	0	
8	2.22387		4.761905	1.646091		25	7.462687	
3.125	1.648805		3.448276	0		0	13.88889	
11.76471	9.803922		13.7931	0		0	50.84746	
18.18182	2.86533		11.11111	1.607717		50	13.1579	
14.28571	2.73224		16.66667	3.225806		0	0	
1.754386	2.912621		1.923077	2.150538		0	10	
7.692307	4.109589		0	3.030303		33.33333	14.28571	
3.571429	1.37931		0	1.503759		25	0	
11.53846	1.369863		0	0.740741		60	9.090909	
7.352941	4.889976		3.278688	2.724796		42.85714	23.80952	
22.98851	5.333333		23.80952	1.538462		22.22222	30	
0.946074	2.247191		1.036269	1.265823		0	10	
10.20408	0.78125		0	0.862069		40	0	
0	6.666667		0	5.405406		0	12.5	
