# Supplementary material for: Defective glutamate and K+ clearance by cortical astrocytes in familial hemiplegic migraine type 2
Source: EMBO Mol Med. 2016 Jun 27;8(8):967–86. doi: 10.15252/emmm.201505944 (PMC4967947; doi:10.15252/emmm.201505944)
Supplement: Supplementary file 9 — Source Data for Figure 5 [file EMMM-8-967-s007.pdf]

Figure 5 Source Data

WT I_K τ_{decay}	KI I_K τ_{decay}
2.40	2.31
2.58	2.68
3.53	2.35
2.35	2.85
1.74	2.81
1.84	3.42
2.66	3.04
1.88	3.13
2.65	2.77
2.69	2.39
2.35	4.17
3.39	2.95
1.95	2.79
1.99	2.75
2.01	3.57
2.52	2.55
2.23	2.96
2.75	2.60
2.04	2.74
2.04	2.53
2.00	
