# Supplementary material for: Defective glutamate and K+ clearance by cortical astrocytes in familial hemiplegic migraine type 2
Source: EMBO Mol Med. 2016 Jun 27;8(8):967–86. doi: 10.15252/emmm.201505944 (PMC4967947; doi:10.15252/emmm.201505944)
Supplement: Supplementary file 10 — Source Data for Figure 6 [file EMMM-8-967-s008.pdf]

Fig 6 Panel B Left Source Data

WT thresh	KI thresh
170	150
310	170
270	150
250	170
230	170
250	150
310	150
270	150
190	150
210	150
250	170
250	170
270	170
270	230
230	190
230	190
210	190
250	150
250	190
190	130
210	150
190	150
190	190
210	170
	210
	190
	190

Fig 6 Panel B Right Source Data

WT velocity	KI velocity
2.89	4.29
3.18	3.70
3.57	4.02
3.36	4.13
3.52	3.70
3.59	4.08
3.45	3.75
3.07	3.79
2.71	3.53
3.09	4.21
3.39	4.03
3.45	3.84
3.19	3.92
3.57	4.13
3.07	3.86
2.89	3.88
3.13	4.00
3.39	3.97
3.68	4.22
3.17	4.19
3.28	3.87
2.96	3.68
3.23	3.72
3.41	3.78
	4.21
	4.23
	3.71
