# Supplementary material for: Defective glutamate and K+ clearance by cortical astrocytes in familial hemiplegic migraine type 2
Source: EMBO Mol Med. 2016 Jun 27;8(8):967–86. doi: 10.15252/emmm.201505944 (PMC4967947; doi:10.15252/emmm.201505944)
Supplement: Supplementary file 11 — Source Data for Figure 7 [file EMMM-8-967-s009.pdf]

Fig 7 Panel A Upper Source Image

Original resolution images (97.5 dpi; generated by Quantity One Chemidoc software)

Precision Plus Protein
Kaleidoscope Standards
(Bio Rad; Catalog #161-0395,
Lot #001649A)

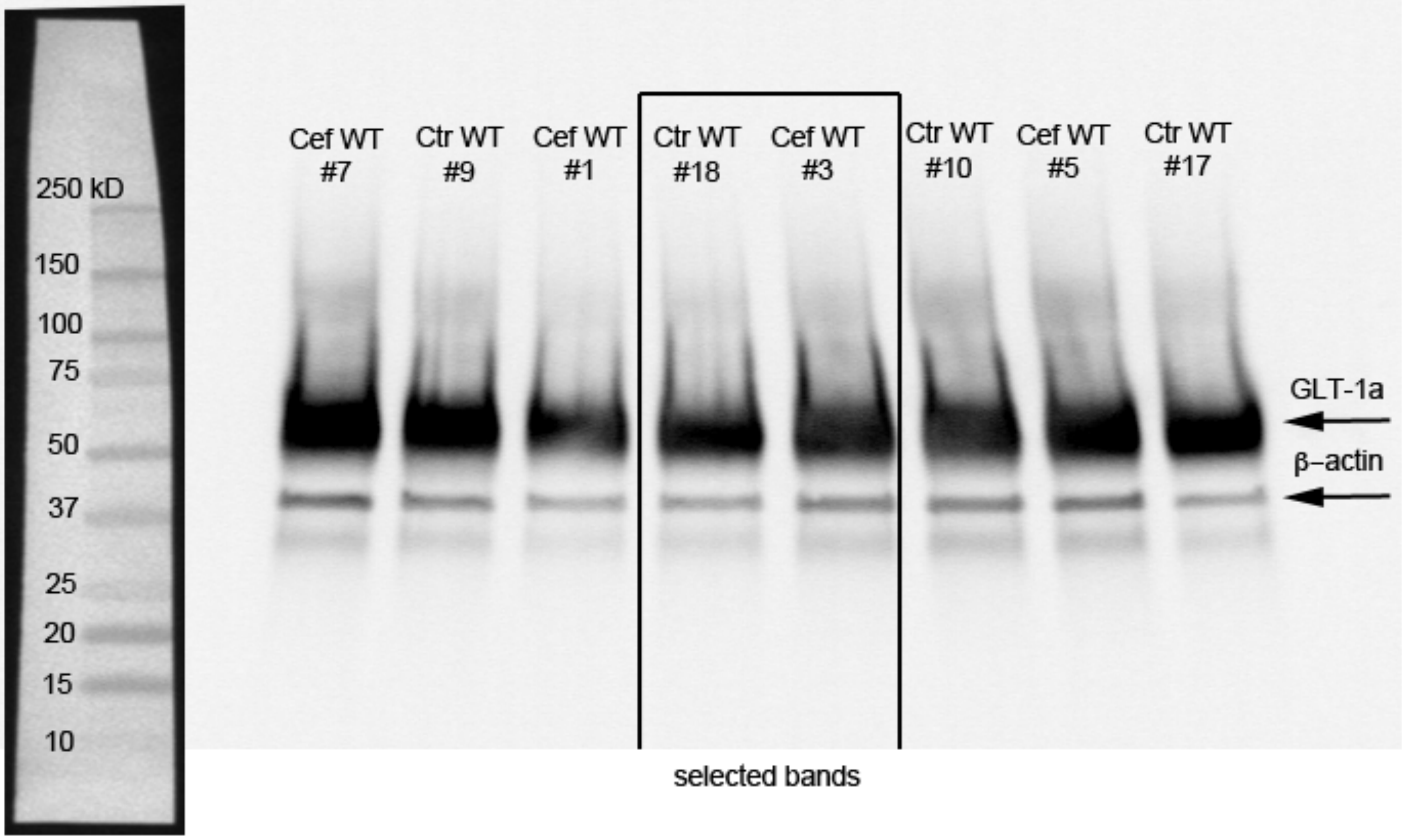

a) After electroblotting column with Kaleidoscope Standards was cut and separated from the nitrocellulose and then acquired by Chemidoc in white mode using Epi White light source. The light 10kD band represents the end point of the electrophoresis run.

b) Each Ctr and Cef case was experimented in 4 gels (see material and methods for details on data analysis)

Fig 7 Panel A Lower Source Data

Ctr WT	Cef WT
89.15663	178.3133
112.6689	175.7576
114.6404	135.1588
83.53414	164.8047

Fig 7 Panel B Left Source Images

original 72 dpi images (color scale method)

Ctr WT | Cef WT

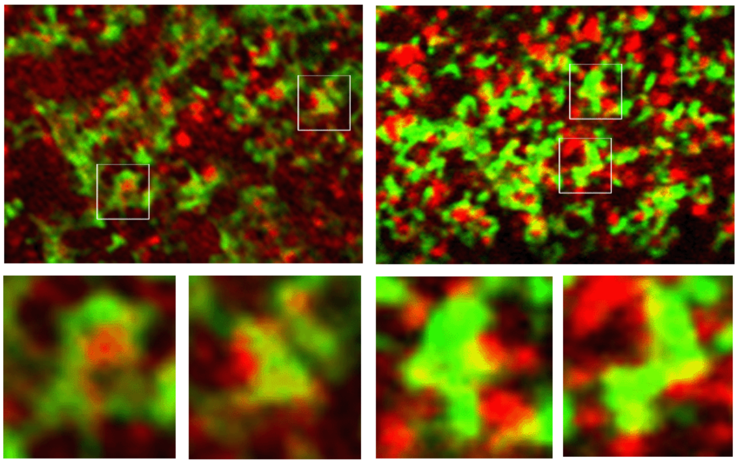

enlarged framed regions

Fig 7 Panel D Right Source Data

Ctr WT			Cef WT		
0.504	0.216	0.4752	1.7136	0.3024	1.296
0.5328	0.432	0.3744	0.2304	1.4112	0.6912
1.4112	1.6416	0.8208	2.0016	0.36	0.6048
0.3312	0.2592	0.2304	0.3888	0.3888	0.864
0.4752	0.2592	0.5328	0.2448	0.4032	0.8208
0.5472	0.3456	0.9792	0.4608	0.6912	0.4752
0.2448	1.7424	0.1728	1.6992	0.36	0.9936
0.6768	0.2736	0.9792	1.6416	0.2736	0.8208
0.1152	0.144	0.2592	1.368	0.648	1.0944
0.3024	0.5184	0.9792	1.6272	0.8352	0.9648
0.288	0.4608	0.3312	2.5488	0.504	0.6768
0.3744	0.3744	0.1584	0.1872	0.6768	0.864
0.4608	0.5472	0.8064	0.3456	0.6048	0.9792
0.4464	0.3456	0.4464	0.2736	0.6048	0.7776
0.1728	0.4176	0.6912	0.1584	0.1296	1.8432
1.872	0.4176	1.08	0.072	0.5328	0.9936
0.4464	0.6624	0.576	0.1152	0.9504	0.9936
3.024	0.3168	0.9792	1.1376	0.3456	0.792
0.0864	0.4464		2.664	0.7344	1.4976
0.1872	0.2592		0.8928	0.4176	1.152
0.2304	0.6192		0.1296	0.5472	1.656
1.2384	0.4464		0.288	0.8208	1.3104
1.0656	0.6624		0.3168	1.1664	0.1872
1.296	0.4896		1.1664	2.1168	0.72
0.7632	0.936		0.7488	0.6192	0.2448
0.3168	0.2016		0.5616	0.7776	0.8064
0.1872	0.1872		0.288	0.5328	1.8576
0.216	0.2304		0.3024	1.9872	1.3392
1.3968	0.4608		1.2816	0.2304	0.8928
0.2304	0.3744		1.3824	0.1872	0.6192
0.3888	0.2016		0.4176	0.8496	0.9792
0.1008	0.1008		0.7632	1.6704	0.4176
0.3168	0.6912		2.376	0.9792	0.4176
0.5328	0.2592		0.9216	1.3104	0.6048
0.648	0.288		1.9584	0.9936	0.3744
0.8352	0.8208		0.4608	1.872	0.2592
0.3168	0.4032		1.0368	0.3168	0.792
0.216	0.2448		0.7056	0.9504	0.7344
0.6336	0.288		2.0736	0.6768	0.4176
0.432	0.648		1.1664	0.792	0.5904
0.3312	0.216		2.5056	0.9936	1.2816
0.216	0.2592		0.7056	0.7056	0.6192
0.6624	0.5184		0.36	0.8928	1.0368
0.6768	0.6768		1.008	1.0368	1.008
0.2592	0.4032		1.4688	0.6768	0.936
0.2448	0.4032		0.5616	0.5904	1.7136
0.5328	0.1728		0.2592	0.8064	1.9296
0.3456	0.36		0.6192	0.8064	1.6416
0.8352	0.36		0.2592	0.7488	0.4608
0.432	0.288		0.72	1.1232	0.864
0.5184	0.1008		0.3888	0.5472	0.3312
0.4896	0.3456		0.576	0.3312	2.0304
1.1376	0.5904		0.5904	1.1376	1.152
0.1728	1.7856		0.3456	1.1232	0.4896
1.0656	0.8496		0.4032	0.216	0.4896
0.4896	0.5328		0.8496	0.8208	0.8208
1.656	0.1728		0.9216	1.2384	1.9728
0.7776	0.144		0.648	0.6912	0.6336
1.2672	0.1872		0.8496	0.8784	0.2592
0.9792	0.3312		1.3248	0.5616	0.5184
0.2016	0.1296		0.8208	0.36	0.4608
0.1296	0.1584		0.288	0.1728	0.5904

Fig 7 Panel C Left Source Data

KI Ctr thresh	KI Cef thresh
130	170
150	210
110	210
110	190
130	150
110	110
150	190
150	170
130	170
170	190
110	150
130	170
150	230
150	170
130	150
150	130
170	170
130	150
150	150
130	190
190	110
90	170
190	110
170	170
150	170
110	150
150	150
150	130
130	130
170	130
190	170
130	
130	
90	
170	
150	
230	
170	

Fig 7 Panel C Right Source Data

KI Ctr velocity	KI Cef velocity
3.88	2.89
3.68	3.56
4.67	3.48
3.39	3.59
3.49	3.64
4.55	4.06
3.77	4.77
3.26	3.49
4.06	4.75
4.10	3.55
3.64	3.56
3.48	4.22
4.63	3.83
4.47	3.25
4.62	4.22
3.40	3.59
4.00	3.69
2.84	3.47
4.07	3.83
3.70	3.48
3.41	3.98
4.18	3.73
3.77	4.81
3.6	3.82
3.82	3.79
4.00	3.52
3.50	3.96
4.28	3.93
3.20	3.93
3.67	3.97
3.65	4.10
3.51	
3.80	
4.08	
4.31	
3.84	
3.85	
3.27	
