# Supplementary material for: Defective glutamate and K+ clearance by cortical astrocytes in familial hemiplegic migraine type 2
Source: EMBO Mol Med. 2016 Jun 27;8(8):967–86. doi: 10.15252/emmm.201505944 (PMC4967947; doi:10.15252/emmm.201505944)
Supplement: Supplementary file 12 — Source Data for Figure 8 [file EMMM-8-967-s010.pdf]

Fig 8 Panel A Left Source Images

original 300 dpi images

Ctr KI

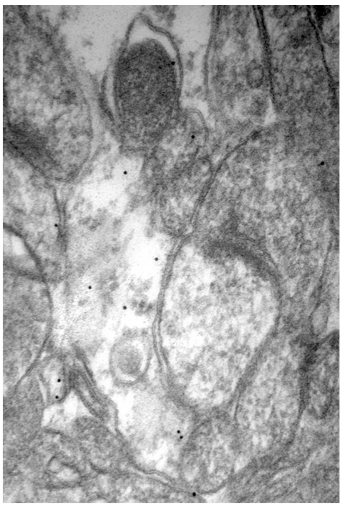

Cef KI

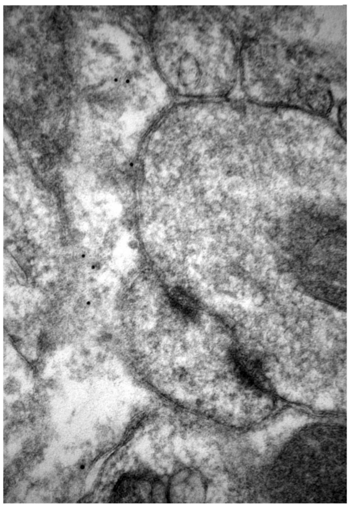

Fig 8 Panel A Right Source Data

Ctr KI

t			cyt			mem		
9.90099	16.66667	21.97802	6.097561	0	14.59854	26.31579	43.47826	44.44444
1.941748	29.06977	19.23077	1.075269	9.259259	18.51852	10	62.5	20
3.131524	32.6087	9.661836	1.190476	42.55319	6.944445	16.94915	22.22222	15.87302
3.816794	10.9489	5.934718	4.716981	4.878049	3.861004	0	28.98551	12.82051
3.623188	17.85714	6.756757	2.405774	0	8.695652	15.03759	58.82353	0
13.33333	14.16431	9.584664	7.092198	9.966778	4	23.80952	38.46154	31.74603
15.625	21.73913	8.982036	22.72727	18.51852	10.48951	0	26.31579	0
3.278688	13.11475	4.716981	2.48139	8.064516	0	9.174312	35.08772	21.73913
2.87687	3.12989	7.407407	1.914486	1.805054	10.98901	11.69591	11.76471	0
2.21533	29.22078	8.547009	1.90295	28.463	0	6.451613	33.70787	28.57143
18.51852	10.60071	12.98701	32.25806	4.255319	22.72727	0	41.66667	0
40	13.1579	7.142857	20.83333	5.988024	0	74.07407	32.78688	22.22222
4.287245	6.779661	15.38461	3.926702	4.444445	0	5.91716	14.28571	31.25
7.692307	10.17812	23.25581	9.803922	3.134796	13.88889	0	40.54054	35.08772
4.504505	11.42857	6.410256	3.838772	7.936508	4.545455	14.70588	20.40816	10.86957
5.617978	25.86207	14.92537	7.462687	38.96104	20.27027	0	0	0
5.698006	16.12903	5.194805	3.401361	27.02703	2.985075	17.54386	0	20
3.931848	7.692307	25.64103	1.494768	12.04819	0	21.2766	0	74.07407
8.230453	11.39601	15.55556	4.862236	6.734007	13.44086	26.78572	37.03704	25.64103
9.077156	4.424779	12.60504	4.761905	0	12.19512	96.77419	14.92537	15.15152
5.24659	9.009009	9.803922	3.476246	12.57862	12.90323	22.22222	0	0
1.939864	44.69274	32	2.239642	44.11765	44.11765	0	46.51163	17.54386
3.6452	28.16901	6.688963	4.184101	37.03704	4.608295	0	22.72727	12.19512
2.159827	19.60784	10.86957	2.55102	27.02703	10.06711	0	0	14.28571
3.246753	25.64103	4.145078	4.048583	25	3.484321	0	27.02703	9.615385
11.40684	18.18182	5.420054	4.62963	34.48276	6.21118	42.55319	0	0
9.90099	16.66667	6.116208	0	0	5.793743	28.57143	35.71429	8.474576
9.489917	6.198347	6.622517	4.021448	2.463054	9.433962	51.54639	25.64103	0
18.75	45.75163	7.017544	18.51852	34.78261	9.615385	19.23077	78.94736	0
1.451379	3.194888	5.067567	0.517331	0	0	14.92537	19.23077	30.61225
8.620689	16.94915	16.39344	0	11.17318	11.49425	44.44444	35.08772	28.57143
9.746589	19.23077	2.12766	11.28668	14.38849	0	0	34.88372	12.5
11.83432	11.97605	12.56432	16.80672	7.633588	13.5495	0	27.77778	21.45344
8.264462	7.017544	10.43457	3.164557	2.217295	12.56566	42.55319	25.21008	22.43546
8	8.797654	13.56643	5.376344	10.90909	14.43456	15.625	0	25.23256
10.98901	54.6875	6.46554	7.352941	40.81633	9.344544	21.73913	63.29114	18.34377
8.281573	7.317073	18.34433	4.784689	5.617978	19.34348	30.76923	18.51852	21.34358
17.24138	23.62205	13.6864	17.3913	31.57895	14.44668	16.94915	0	18.34383
6.802721	21.50538	12.54468	4.081633	46.51163	13.55466	20.40816	0	15.23423
34.48276	16.2037	19.44645	76.92308	14.08451	21.45657	0	25.97403	0
6.837607	10	18.12436	4.040404	25	20.33344	22.22222	0	20.32323
2.887392	16.31702	6.434343	1.067236	17.14286	9.232357	19.60784	12.65823	21.33437
2.070393	30.30303	4.906655	0.773994	52.63158	6.676544	12.73885	0	18.43855
6.337136	18.69159	18.45458	5.797101	12.98701	20.76764	10.10101	33.33333	19.43869
6.359301	23.12139	19.24323	5.347594	0	21.65654	14.70588	71.42857	40.43478
3.846154	6.153846	20.34353	4.926108	3.846154	23.65653	0	15.38461	13.43435
23.52941	17.24138	12.54545	8.264462	14.70588	14.45457	61.22449	20.83333	18.43476
7.662835	18.34862	25	7.786429	29.41176	27.34543	6.896552	0	3.656585
5.758157	16.52892	13.34344	2.631579	12.82051	15.34343	14.1844	23.25581	23.45857
4.464286	27.02703	12.22424	2.673797	22.22222	13.94876	13.51351	34.48276	10.45485
2.580645	8.264462	10.35434	1.37931	13.1579	11.37175	20	0	9.383843
7.77605	17.64706	11.32434	5.545287	17.69912	12.48672	19.60784	17.54386	20.43758
5.044136	8.064516	13.43244	1.410437	10.92896	14.34384	35.71429	0	21.32376
50	9.803922	10.23232	166.6667	14.49275	12.34373	0	0	0
4.983389	9.389671	9.345344	3.745318	6.079027	11.49869	14.70588	20.61856	25.34386
9.009009	88.23529	8.232323	7.490637	153.8462	10.48576	15.15152	47.61905	13.54868
5.617978	6.849315	7.323235	6.514658	0	8.343848	0	18.51852	11.43846
6.31579	18.93939	6.223454	2.409639	14.42308	9.549574	33.33333	35.71429	20.32876
13.51351	6.637168	12.13456	5.91716	0	13.96844	37.73585	37.03704	21.43896
2.284844	18.01802	16.34343	2.504174	12.82051	19.48668	0	30.30303	20.43434
3.198294	5.681818	15.98977	2.293578	13.1579	18.54548	15.15152	0	12.45455
4.555809	3.88601	13.98878	5.249344	4.754358	15.4394	0	0	18.45446
3.144654	25	12.87765	3.846154	21.97802	14.76848	0	34.48276	21.43443
18.18182	40.32258	13.78676	0	34.88372	15.55832	66.66666	52.63158	19.45469
34.09091	11.08033	12.34344	47.61905	6.279435	14.69831	0	47.05882	13.43434
6.666667	9.836065	11.93844	8.130081	7.633588	13.67946	0	23.25581	15.76655
12.19512	32	10.34346	7.692307	13.33333	12.65657	29.41176	60	18.34534
2.638522	10.20408	9.337344	3.154574	16.94915	11.67944	0	0	20.43432
4.123711	23.25581	8.232738	3.658537	12.82051	10.56868	6.666667	39.21569	21.23256
4.250797	9.259259	15.34343	3.605769	10.72961	17.33431	9.174312	0	21.65656
1.873829	31.74603	16.34346	2.054795	33.33333	18.58712	0	30.30303	279.4545
2.989537	6.060606	13.45565	3.496504	7.936508	15.39044	0	0	40.34323
1.988072	19.23077	18.34345	0	0	20.45957	10.6383	50	23.42334
4.518072	11.90476	12.45455	5.328597	7.352941	14.55434	0	31.25	18.34538
5.474452	18.07229	25.67654	2.237136	15.09434	26.43333	19.80198	29.85075	30.52326
3.278688	5.773672	4.656558	1.834862	6.570302	0	15.38461	0	20.43141
9.174312	19.70443	3.659859	0	18.18182	2.43433	29.19708	26.31579	4.645324
3.948667	12.04819	40.32323	2.361275	7.874016	2.323232	12.04819	25.64103	60.43436
2.95858	5.044136	3.654985	2.171553	2.836879	4.323226	10.75269	22.72727	18.42424
2.20022	21.58273	7.454939	2.427185	23.52941	8.343433	0	18.51852	10.43525
1.633987	9.259259	12.4596	1.834862	14.08451	10.34348	0	0	33.64645
9.404388	10.6383	16.44935	8.403361	14.28571	14.23486	12.34568	0	19.44242
3.154574	9.174312	15.76764	0	0	12.5439	17.54386	38.46154	18.42424
1.207729	26.08696	18.43545	0	22.98851	15.32326	6.802721	35.71429	18.42422
6.568144	8.298756	20.54545	1.923077	5.649717	18.23423	33.70787	15.625	21.33533
1.52439	8.77193	21.34346	0	0	17.34398	14.59854	29.41176	24.53533
2.259036	16.18123	13.76768	1.793722	20.24291	11.32985	4.694836	0	15.22544
4.731861	86.95652	10.56566	0	0	8.348439	36.14458	90.90909	13.53433
1.213592	8.403361	9.565555	0	14.49275	11.33433	9.345795	0	13.53531
1.422475	7.434944	10.65657	0	8.888889	8.434336	11.76471	0	13.53534
3.194888	18.69159	7.343432	3.773585	0	5.232676	0	33.8983	20.67465
7.905138	24.59016	6.343436	5.524862	24.39024	3.575422	13.88889	25	23.64665
3.427592	19.84127	5.676764	1.926782	16.48352	2.545488	15.50388	28.57143	15.65634
2.483444	50.63291	4.656545	1.831502	27.77778	2.434745	8.620689	69.76744	13.57846
3.600823	23.52941	17.34343	2.816901	17.54386	12.34344	11.83432	35.71429	19.45454
4.524887	11.11111	13.65656	3.021148	0	10.32439	13.45292	31.25	26.42422
4.467134	16.66667	12.45455	2.148997	10.98901	10.43867	23.39181	34.48276	22.42147
1.930502	10.52632	11.65656	1.414427	7.8125	9.646545	7.142857	16.12903	28.44255
3.666361	4.716981	10.34344	1.108525	5.681818	8.343212	15.88142	0	13.32313
0.311527	11.76471	11.65656	0.333333	0	12.43438	0	28.98551	14.64647
1.03146	5.235602		0.579039	3.184713		4.716981	14.70588	
9.404388	17.54386		7.968128	12.5		14.70588	29.41176	
1.907184	55.55556		0.707714	95.2381		12.5	0	
5.016722	21.89781		2.873563	19.5122		19.73684	28.98551	
12.44813	9.708738		5.91716	12.16545		27.77778	0	
3.703704	22.47191		0	0		21.42857	83.33334	
8.264462	9.803922		3.311258	15.15152		32.78688	0	
4.219409	11.04972		2.009377	11.02941		24.09639	11.11111	
2.976191	11.23596		0.881057	17.85714		14.35407	0	
3.636364	11.56069		0	6.734007		17.24138	40.81633	
5.274261	46.72897		2.594034	53.33333		16.94915	31.25	
12.76596	10.20408		10.30928	0		24.39024	22.22222	
6.134969	23.72881		0	22.42152		19.48052	27.77778	
57.14286	6.369427		58.82353	0		56.60378	18.86792	
4.016064	40.57971		2.457002	38.7324		10.98901	49.18033	
7.782101	24.39024		5.181347	12.73885		15.625	62.5	
1.461988	7.874016		0	0		13.51351	26.31579	
14.08451	22.85714		0	18.69159		61.22449	14.70588	
5.369128	23.25581		3.284072	25.31646		14.70588	20	
14.53488	2.976191		7.142857	3.521127		46.875	0	
5.291005	11.90476		0	3.610108		24.59016	33.8983	
21.69422	17.44186		22.51185	15.03759		16.12903	25.64103	
20.20202	16.85393		19.48052	15.87302		22.72727	21.50538	
17.3913	25		13.33333	18.86792		25	37.03704	
10.10101	17.69912		7.352941	12.34568		16.12903	31.25	
33.70787	24.59016		34.48276	34.09091		32.25806	0	
11.17318	29.85075		0	25		33.8983	37.03704	

Cef KI

t			cyt			mem		
17.24138	2.890173	4.016064	0	3.584229	0	26.31579	0	27.39726
5.434783	15.93626	26.31579	3.164557	21.62162	16.66667	19.23077	0	62.5
18.56149	6.485085	15.69507	21.14804	5.714286	10.6383	10	14.08451	42.85714
1.485884	3.861004	7.561437	0	0	4.469274	7.751938	12.98701	24.53988
4.106776	5.479452	23.68421	2.631579	4.545455	19.23077	9.345795	14.28571	33.33333
4.048583	23.07692	36.14458	3.220612	11.36364	39.21569	8.333333	47.61905	31.25
13.58234	2.283105	18.51852	9.960159	1.629992	25.21008	34.48276	11.49425	0
10.69519	19.73684	12.82051	3.08642	18.18182	7.936508	60	23.80952	33.33333
60.60606	9.55414	98.03922	100	3.787879	37.03704	0	40	166.6667
7.619048	5.657709	85.10638	4.301075	3.384095	52.63158	33.33333	17.24138	107.1429
9.708738	35.71429	15.46392	7.608696	0	4.310345	27.27273	52.63158	59.32203
8.379889	50.84746	7.326007	11.11111	51.28205	4.926108	0	50	14.28571
3.846154	7.352941	12.5	0	7.153076	8	11.11111	8.547009	28.57143
14.92537	3.267974	7.8125	10.41667	1.813237	0	26.31579	16.52892	34.48276
17.09402	28.98551	4.293893	11.36364	22.22222	3.205128	34.48276	41.66667	13.39286
15.6658	24.09639	14.74926	18.46154	51.28205	10.98901	0	22.72727	30.30303
24.75248	28.16901	12.2164	30.76923	22.22222	12.12121	13.88889	38.46154	12.82051
3.571429	13.88889	32.31018	4.545455	0	14.52282	0	38.46154	94.89051
7.017544	15.625	8.667389	8.163265	0	7.566204	0	57.14286	15.38461
21.05263	13.51351	36.42384	15.38461	18.51852	24.27184	33.33333	0	62.5
7.438016	7.874016	13.47305	6.542056	0	7.380074	14.28571	36.36364	39.68254
3.463203	17.75148	50.63291	1.932367	8.695652	39.21569	16.66667	37.03704	71.42857
5	19.80198	14.32958	6.122449	13.69863	11.2782	0	35.71429	27.93296
6.122449	5.681818	2.994012	8.695652	1.290323	3.597122	3.846154	38.09524	0
3.846154	5.163511	8.097166	4.545455	4.08998	5.050505	0	10.86957	20.40816
1.769912	4.494382	55.55556	2.222222	5.449591	0	0	0	90.90909
6.224066	9.646302	14.0056	5.076142	4.608295	12.15278	11.36364	21.2766	21.73913
5.221023	5.780347	6.525285	4.868914	7.874016	5.381166	9.852217	0	18.01802
2.054795	23.52941	5.736138	2.290076	25	6.622517	0	22.22222	0
6.382979	2.962963	18.40491	4.032258	3.683241	16.46091	23.52941	0	24.09639
7.954545	8.264462	18.60465	7.792208	0	12.34568	9.090909	27.02703	37.73585
4.878049	13.33333	12.00686	5.797101	0	5.813953	0	33.33333	59.70149
2	3.937008	8	1.515152	0	5.649717	5.555555	27.39726	13.69863
4.347826	3.816794	14.81481	0	4.830918	11.62791	16.66667	0	20.40816
34.24657	12.04819	28.57143	37.03704	0	20.40816	30.76923	41.66667	47.61905
20.20202	10.27397	7.537688	15.15152	8.474576	6.472492	30.30303	17.85714	11.23596
31.25	7.425743	18.86792	45.45454	8.450705	25.31646	0	0	0
14.28571	7.246377	5.813953	8.333333	12.98701	7.518797	27.27273	0	0
10.34483	5.012531	6.662225	5	3.154574	2.941176	22.22222	12.19512	42.55319
8.333333	1.369863	23.19588	11.11111	3.333333	13.51351	0	0	54.34783
22.90076	2.183406	22.38806	23.52941	0	20.20202	21.73913	11.82033	28.57143
16.94915	2.295333	5.208333	27.02703	1.668057	3.10559	0	9.259259	16.12903
15.22843	6.079027	8.583691	7.29927	3.691399	6.802721	33.33333	17.2117	11.62791
6.578948	11.11111	4.22833	8.333333	9.259259	3.554502	0	13.88889	9.803922
15.38461	5.830904	5.540166	14.28571	7.079646	1.533742	20	0	42.85714
17.24138	5.357143	4.310345	50	3.896104	0	0	21.42857	24.39024
23.91304	100	2.994012	17.64706	0	2.616431	41.66667	214.2857	10.75269
37.38318	2.008032	7.594937	42.55319	2.460025	5.952381	33.33333	0	16.94915
4.615385	2.109705	10.10101	1.754386	0	4.761905	25	11.11111	40
11.53846	11.11111	25.90674	5.263158	6.944445	20.97902	28.57143	27.77778	40
13.84615	26.31579	4.524887	9.259259	0	6.578948	36.36364	95.2381	0
14.92537	4.310345	4.694836	0	5.681818	0	31.25	0	16.12903
26.45503	103.4483	9.615385	28.77698	138.8889	2.375297	20	45.45454	40.40404
7.846556	17.09402	7.125891	7.028112	15.15152	107.1429	13.24503	19.60784	0
7.29927	22.1328	14.88095	5.452563	22.67002	7.692307	16.75978	20	39.47368
15.87302	8.403361	3.215434	13.98601	0	1.828154	21.73913	36.36364	13.33333
13.69863	12.94498	43.47826	28.57143	4.273504	83.33334	0	40	0
14.56311	1.725129	2.877698	12.65823	1.27959	3.336113	20.83333	5.681818	0
5.194805	11.62791	3.636364	2.898551	7.168459	2.69179	25	30.76923	12.19512
6.589786	11.90476	4.098361	3.976143	9.708738	4.524887	19.23077	15.38461	0
26.08696	3.472222	7.168459	5.263158	0	9.569378	125	9.708738	0
12	9.523809	7.220217	5	17.24138	4.424779	40	0	19.60784
12.84109	2.45098	3.773585	9.689922	0	4.291845	28.03738	9.174312	0
3.257329	26.84564	17.3913	0	26.08696	0	8.403361	29.41176	57.14286
12.19512	26.78572	4.608295	8.823529	14.08451	2.617801	28.57143	48.78049	19.23077
22.47191	29.85075	3.170828	16.66667	21.50538	1.26369	34.48276	48.78049	33.55705
25.64103	14.08451	5.110733	37.03704	0	4.716981	0	28.57143	8.77193
6.451613	15.625	2.634352	4	0	2.245929	16.66667	71.42857	8.547009
16.21622	3.968254	17.64706	3.448276	5.882353	8.547009	62.5	0	37.73585
5.882353	4.301075	5.319149	5.084746	2.597403	2.012072	11.11111	12.5	29.85075
7.936508	9.049774	8.928572	5.357143	0	8.474576	28.57143	35.71429	11.36364
5.172414	14.08451	2.376238	0	11.36364	1.738375	42.85714	18.51852	8.928572
6.521739	18.51852	4.296455	2.777778	16	3.663004	20	27.02703	8.928572
9.090909	5.643341	8.526188	5.555555	3.989362	6.868132	25	14.92537	21.50538
61.53846	6.060606	5.422994	55.55556	0	4.83676	68.96552	27.77778	10.52632
18.98734	4.62963	2.171553	25	5.882353	1.207729	0	0	10.75269
16.66667	4.484305	3.162889	16.66667	3.533569	2.339181	16.66667	9.708738	10.69519
15.38461	14.70588	8.21018	0	20.20202	9.708738	37.03704	0	0
4.347826	4.457652	5.330491	5.555555	3.442341	3.584229	0	10.86957	19.80198
6.451613	4.282655	3.521127	4	2.73224	0	16.66667	9.90099	25.97403
6.25	12.01201	6.036217	0	7.352941	3.673769	25.64103	32.78688	30.76923
5.454545	6.521739	14.81481	4.255319	2.518892	4.608295	12.5	31.74603	56.60378
18.86792	2.994012	18.90756	14.08451	0	21.42857	28.57143	14.92537	0
6.666667	4.215852	22.98851	0	2.819549	8.849558	25	16.39344	49.18033
3.192338	2.60983	11.90476	2.690583	1.454898	0	7.246377	12.65823	40.81633
5.285412	6.188119	5.934718	3.512881	4.817619	3.584229	21.73913	18.40491	17.24138
23.52941	12.30769	1.926782	17.54386	11.19403	2.045687	35.71429	17.54386	0
16.66667	5.509642	4.081633	0	0	2.793296	50	28.57143	13.24503
14.81481	5.870842	1.121705	15	0	1.196888	14.28571	23.62205	0
12.12121	8.450705	10.75269	8.333333	10.30928	7.832898	22.22222	0	24.39024
54.26357	3.846154	9.708738	66.66666	4.784689	7.8125	37.03704	0	18.86792
8.108109	4.225352	2.762431	3.571429	4.815409	3.267974	22.22222	0	0
23.80952	5.91716	11.0742	21.42857	7.29927	8.578431	28.57143	0	34.48276
5.405406	6.430868	4.065041	5.263158	3.90625	1.602564	6.25	18.18182	17.54386
9.523809	5.905512	11.17318	6.666667	4.62963	3.717472	16.66667	13.1579	33.70787
9.090909	8.264462	4.205215	8.333333	0	2.020202	11.11111	27.02703	15.07538
12.82051	16.48352	10.9489	8.333333	30.61225	8.350731	45.45454	0	28.98551
48.07692	2.123142	7.707129	55.55556	1.213592	4.576659	31.25	8.474576	24.39024
3.389831	1.686341	3.415884	2.803738	2	1.863933	9.090909	0	20.40816
22.38806	2.980626	6.535948	25.97403	1.692047	0	17.54386	12.5	26.31579
28.57143	41.66667	4.666667	30.30303	0	4.428044	23.25581	81.08108	6.896552
12.42236	3.831418	5.141388	15.5642	0	6.060606	0	25	0
4.237288	7.005254	5.471956	0	6.382979	3.189793	23.80952	9.90099	19.23077
27.39726	6.802721	4.854369	32.25806	8.849558	0	23.80952	0	16.66667
20.97902	6.269592	7.566204	18.69159	3.846154	5.763689	27.77778	16.94915	20.20202
66.66666	16.85393	10.08249	66.66666	0	8.298756	66.66666	57.69231	23.62205
22.72727	42.68293	5.05476	0	50	5.591799	48.78049	22.72727	0
3.968254	23.25581	13.7931	0	0	13.04348	17.85714	62.5	16.66667
16.80672	3.210273	10.12146	20.30457	0	9.280743	0	24.69136	15.87302
24.39024	12.5	12.57862	24.19355	6.369427	0	25	24.09639	66.66666
30.30303	2.572899	9.925558	0	0.987167	5.882353	74.07407	13.0719	31.74603
19.73684	3.081664	1.808972	12.98701	1.848429	1.552192	26.66667	9.259259	5.347594
15.15152	4.048583	17.75148	0	2.314815	8.064516	28.57143	16.12903	44.44444
44.77612	40	10.67073	55.55556	111.1111	5.244755	32.25806	0	47.61905
7.874016	2.123142	9.049774	13.88889	0	10.92896	0	7.518797	0
25.64103	8.695652	18.69159	58.82353	0	12.65823	0	31.25	35.71429
22.72727	24.39024	8.196721	23.07692	0	3.584229	21.73913	47.61905	22.98851
16.44737	4.87013	8.080808	12.71186	5.882353	7.142857	29.41176	0	13.33333
21.64502	3.913894	24.39024	19.23077	2.235469	23.52941	26.66667	15.70681	27.39726
17.3913	12.37964	9.852217	13.69863	10.76923	8.645534	23.80952	25.97403	16.94915
7.177033	35.29412	4.761905	6.21118	22.22222	2.857143	10.41667	50	23.80952
3.460208	20.40816	4.21456	2.074689	10.75269	3.34275	10.41667	37.03704	13.84015
2.503129	7.092198	7.772021	1.386963	8.849558	3.08642	12.82051	0	32.25806
18.40491	4.032258	11.45038	8.474576	3.424658	9.259259	44.44444	8.620689	21.73913
6.622517	5.069708	16.62708	9.523809	2.932551	6.493506	0	18.69159	44.24779
6.944445	10.84599	13.26367	9.090909	10.55409	10.5197	0	12.19512	23.31032
6.472492	60.60606		0	0		23.52941	117.6471	

Fig 8 Panel B Left Source Images

Ctr KI

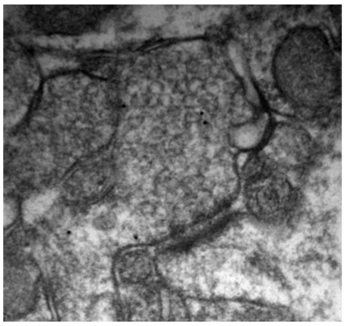

Cef KI

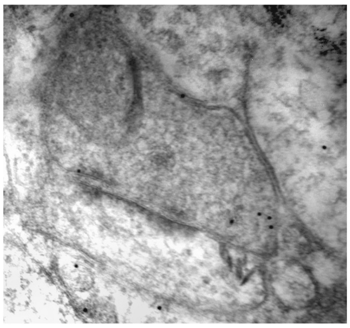

Fig 8 Panel B Right Source Data

Ctr KI

t			cyt			mem		
11.90476	3.076923	2.074689	7.633588	0	2.352941	27.02703	20.40816	0
2.159827	1.30719	22.72727	0	0	0	17.85714	12.82051	62.5
2.886003	1.189061	5.089058	3.184713	0	5.882353	0	13.88889	0
7.407407	7.874016	7.042253	9.615385	3.527337	4.149378	0	44.11765	23.25581
1.119821	3.466204	4.207573	0	1.937984	0	12.65823	16.39344	41.66667
1.422475	1.766784	6.389776	0	0	3.703704	15.15152	15.15152	23.25581
5.16129	1.808318	8.733624	4.273504	2.04918	0	13.69863	0	55.55556
5.263158	7.633588	4.016064	0	0	0	25.64103	33.33333	34.48276
2.941176	5.597015	4.83871	1.650165	4.395605	3.571429	13.51351	12.34568	16.66667
2.105263	2.066116	4.132231	2.398082	2.320186	1.52439	0	0	28.57143
2.192982	12.34568	2.645503	2.48139	10.20408	0	0	21.2766	19.60784
4.255319	4.081633	8.695652	0	0	0	23.80952	37.03704	38.46154
3.603604	6.637168	3.787879	4.024145	6.105006	0	0	11.76471	23.80952
10.05025	1.253133	4.385965	12.04819	0	5.208333	0	14.49275	0
5.649717	3.846154	1.30719	3.424658	4.310345	1.43472	16.12903	0	0
10.90909	4.444445	1.298701	8.510638	1.636661	0	25	31.25	15.15152
5.333333	2.754821	7.672634	2.989537	0	5.91716	24.69136	19.23077	18.86792
10.98901	8.658009	2.941176	14.70588	5.263158	0	0	48.78049	19.23077
8.368201	5.628518	2.386635	4.926108	4.197272	2.73224	27.77778	17.69912	0
14.15094	5.730659	15.32567	0	3.322259	13.69863	50.84746	20.83333	23.80952
3.831418	1.355014	3.024194	2.141328	0	2.188184	18.18182	15.15152	12.82051
4.081633	2.120891	3.278688	4.901961	2.32288	0	0	0	21.73913
3.333333	3.663004	2.670227	3.952569	4.310345	2.932551	0	0	0
3.731343	9.523809	1.867414	4.329004	0	1.017294	0	40	11.36364
4.950495	2.089864	6.329114	3.04878	2.267574	7.352941	13.1579	0	0
10.86957	1.410437	3.30033	12.87554	0	3.846154	0	14.92537	0
5.464481	7.556675	2.212389	6.849315	2.985075	1.20048	0	32.25806	14.08451
12.98701	3.058104	8.310249	17.09402	3.597122	6.369427	0	0	21.2766
3.623188	4.819277	2.525253	0	2.739726	0	19.60784	20	20
1.28041	5.338078	2.631579	1.453488	5.940594	0	0	0	20.83333
3.597122	5.309734	15.15152	4.184101	1.976285	9.569378	0	33.8983	36.36364
2.141328	5.076142	1.6	2.424242	0	1.792115	0	28.57143	0
2.259887	2.312139	5.270092	2.466091	2.541296	4.373178	0	0	13.69863
7.575758	8	1.519757	0	10.10101	1.675042	29.41176	0	0
3.284072	6.134969	1.766784	1.858736	7.092198	0	14.08451	0	17.24138
4	9.090909	4.016064	4.878049	5.524862	4.830918	0	25.64103	0
3.929273	8.583691	2.386635	2.257336	5.076142	2.747253	15.15152	27.77778	0
3.546099	5.730659	1.019368	3.929273	3.322259	0	0	20.83333	13.51351
5.263158	6.557377	2.688172	0	7.633588	1.47929	30.30303	0	14.70588
5.509642	1.934236	5.235602	6.309148	2.164502	6.289308	0	0	0
2.597403	6.060606	15.15152	3.021148	7.017544	4.424779	0	0	78.94736
2.688172	4.166667	4.132231	0	4.975124	2.309469	16.12903	0	19.60784
10.98901	1.824818	14.1844	0	0	17.54386	40	17.85714	0
3.267974	4	4.705883	0	2.247191	2.659575	18.51852	18.18182	20.40816
3.597122	1.605136	4.192872	4.201681	1.769912	4.705883	0	0	0
3.773585	1.724138	3.717472	4.464286	1.912046	3.724395	0	0	0
8.498584	1.386963		10	0		0	14.49275	
3.496504	3.04414		0	0		28.98551	30.76923	
22.09945	3.752345		13.51351	2.10084		60.60606	17.54386	
12.60504	3.550296		15.15152	3.88601		0	0	
3.322259	7.246377		0	8.547009		22.22222	0	
3.759398	4.926108		0	5.540166		18.86792	0	
2.155172	8.77193		2.487562	0		0	38.46154	
1.278772	3.378378		1.408451	3.802281		0	0	
2.087683	7.263923		1.141553	5.509642		12.19512	40	
5.747742	5.376344		3.692647	6.153846		14.50163	0	

Cef KI

t			cyt			mem		
3.225806	9.661836	23.21428	3.731343	2.785515	20.40816	0	54.54546	42.85714
1.594896	8.042895	4.22833	1.788909	3.174603	4.938272	0	34.48276	0
2.832861	9.174312	4.123711	3.257329	5.524862	2.34192	0	27.02703	17.24138
6.269592	3.992016	1.485884	7.29927	2.232143	1.686341	0	18.86792	0
4.662004	2.20022	14.25662	2.65252	1.193317	13.82488	19.23077	14.08451	17.54386
6.493506	8.196721	3.484321	3.891051	0	0	19.60784	48.78049	19.60784
3.984064	2.272727	11.58301	4.694836	0	13.95349	0	19.60784	0
3.937008	4.106776	5.449591	2.207505	0	3.194888	18.18182	31.25	18.51852
2.415459	4.477612	3.236246	2.762431	1.644737	3.745318	0	32.25806	0
6.21118	4.415011	3.667482	4.739336	2.525253	1.351351	16.39344	17.54386	25.64103
10.41667	5.509642	7.874016	6.410256	3.034901	9.036144	27.77778	29.85075	0
6.71141	4.587156	6.198347	3.921569	3.754693	4.640371	23.25581	13.69863	18.86792
11.21076	3.289474	5.221932	10.75269	1.824818	5.970149	13.51351	16.66667	0
3.929273	5.910165	5.725191	2.232143	6.459949	4.291845	16.39344	0	17.24138
9.132421	3.30033	6.396588	5.434783	1.865672	2.43309	28.57143	14.28571	34.48276
11.33144	3.795066	1.190476	6.493506	2.242152	0	44.44444	12.34568	13.51351
7.751938	5.194805	2.702703	0	2.853067	0	44.44444	28.98551	12.65823
4.878049	2.832861	3.112033	0	3.257329	2.355713	29.41176	0	8.695652
2.145923	1.369863	3.516999	2.427185	1.503759	1.293661	0	0	25
3.703704	2.034588	1.22399	0	2.207505	1.362398	23.80952	0	0
6.514658	3.90625	4.819277	3.846154	2.853067	2.840909	21.2766	14.92537	15.87302
1.233046	7.092198	5.376344	0	8.403361	6.578948	14.08451	0	0
19.48052	10.98901	7.968128	22.81369	8.928572	5.076142	0	20.40816	18.51852
4	3.198294	2.28833	2.320186	1.182033	0	14.49275	21.73913	17.85714
7.462687	5.291005	1.615509	4.651163	0	0	18.86792	34.48276	14.08451
3.584229	17.69912	3.539823	0	15.95745	2.083333	25	26.31579	11.76471
13.45292	6.960557	8.982036	5.681818	5.571031	7.142857	42.55319	13.88889	18.51852
3.102379	9.966778	2.673797	2.257336	11.58301	3.174603	12.34568	0	0
6.25	30.92784	7.342144	2.380952	41.09589	4.878049	33.33333	0	30.30303
5.617978	13.11475	4.962779	3.267974	4.201681	2.777778	20	44.77612	23.25581
12.65823	5.703422	3.444317	9.950249	6.535948	2.534854	27.77778	0	12.19512
2.057613	2.773925	2.936857	1.119821	1.567398	0	12.65823	12.04819	29.41176
4.329004	2.237136	13.46801	3.194888	0	8.130081	14.92537	18.51852	39.21569
3.448276	14.88095	6.006006	0	3.472222	0	30.30303	83.33334	40
4.076087	5.509642	1.669449	1.49925	6.688963	0	28.98551	0	15.625
5.434783	13.98601	5.102041	2.028398	9.523809	0	33.8983	26.31579	37.5
3.412969	12.39669	3.813155	1.24533	5.050505	1.037344	26.31579	45.45454	35.29412
67.79661	7.692307	9.259259	102.5641	9.345795	11.17318	0	0	0
2.645503	7.462687	3.944773	3.076923	10.20408	4.535147	0	0	0
5.347594	8.130081	3.629764	0	10.86957	2.04499	31.25	0	16.12903
10.86957	9.463722	9.966778	4.329004	3.703704	4.016064	44.44444	42.55319	38.46154
4.846527	6.19195	4.77707	5.376344	3.717472	3.565062	0	18.51852	14.92537
2.713704	4.366812	3.033367	3.012048	1.672241	1.102536	0	22.47191	24.39024
1.28041	9.708738	7.518797	0	0	4.464286	14.08451	57.14286	23.80952
4.132231	5.494505	6.389776	2.336449	6.756757	3.831418	17.85714	0	19.23077
7.707129	12.76596	4.231312	6.493506	5.208333	1.560062	17.54386	46.51163	29.41176
1.146395	5.617978	7.407407	1.256281	2.087683	4.444445	0	36.36364	22.22222
7.874016	8.849558	7.772021	0	6.369427	5.899705	50	14.49275	21.2766
2.070393	6.21118	2.55102	2.325581	5.172414	2.976191	0	15.625	0
1.996008	3.058104	6.024096	2.237136	3.546099	4.640371	0	0	14.92537
3.875969	16.26016	6.437768	0	10.98901	4.987531	45.45454	31.25	15.38461
6.23053	3.125	3.916449	7.490637	3.539823	1.461988	0	0	24.39024
7.662835	3.937008	4.106776	4.878049	0	0	17.85714	24.39024	36.36364
4.683841	20.40816	3.430532	2.645503	19.73684	0	20.40816	22.72727	32.78688
9.132421	3.584229		11.97605	2.061856		0	13.69863	
4.944376	12.19512		5.41272	10.56338		0	22.72727	

Fig 8 Panel C Upper Source Image

Original resolution images (97.5 dpi; generated by Quantity One Chemidoc software)

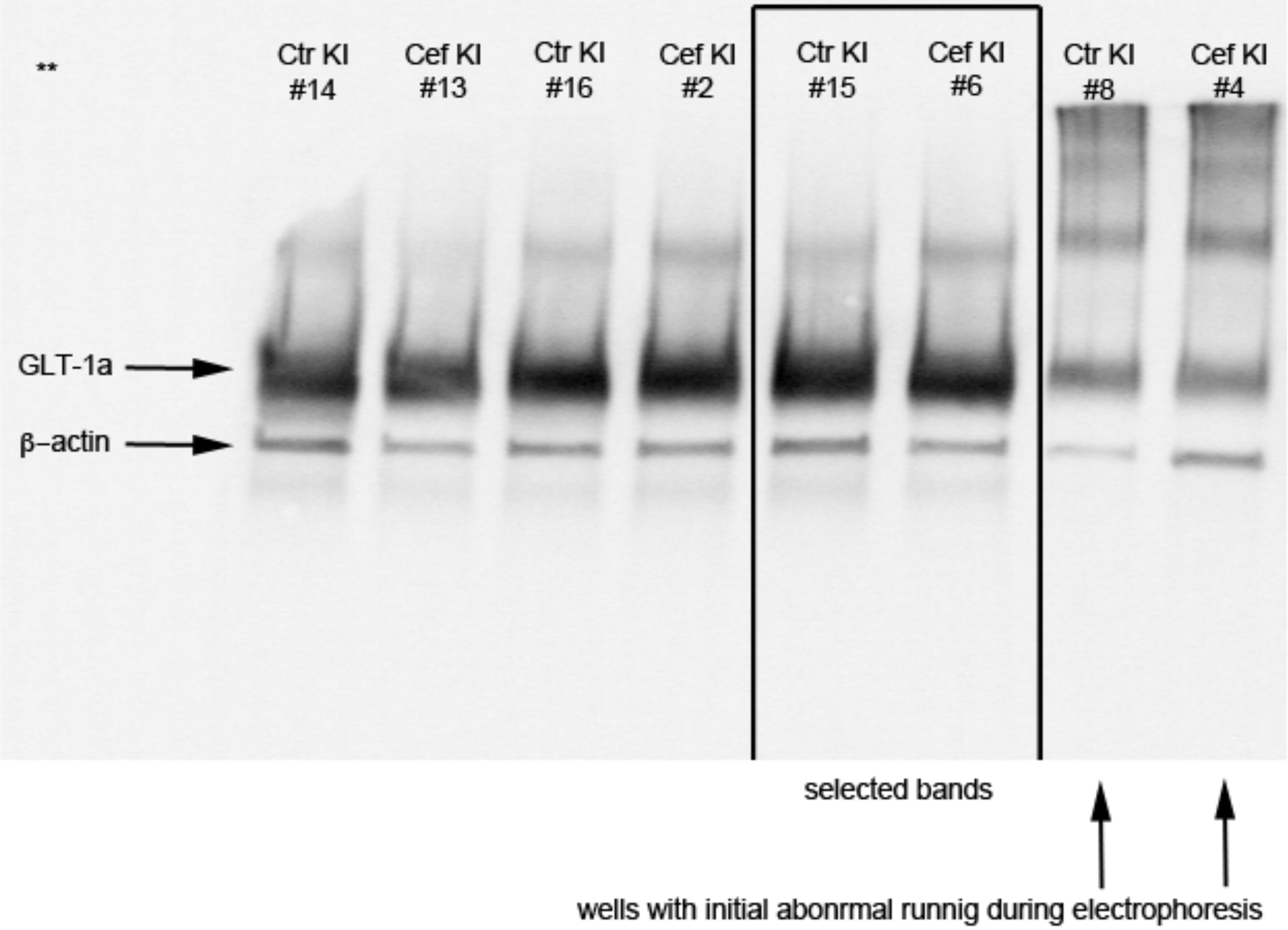

a) **Given that GLT-1a and β–actin molecular weights were verified in WT experiments, Kaleidoscope Standards were not loaded

b) Each Ctr and Cef case was experimented in 6 gels (see material and methods for details on data analysis)

Fig 8 Panel C Lower Source Data

Ctr KI	Cef KI
84.41247	118.9582
129.7762	102.2649
81.30829	119.7309
104.5031	84.19931

Fig 8 Panel D Left Source Images

original 72 dpi images (color scale method)

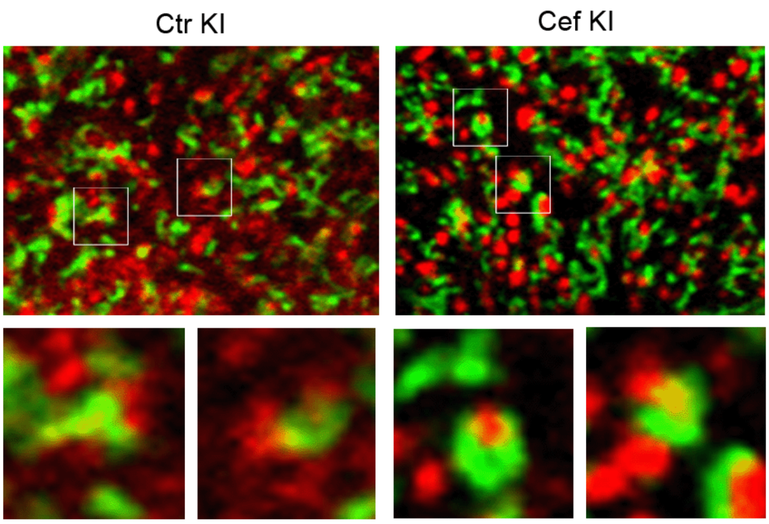

enlarged framed regions

Fig 8 Panel D Right Source Data

Ctr KI			Cef KI		
0.1728	0.1584	0.504	0.2592	0.6192	1.0368
1.0368	0.9216	0.1584	0.5184	0.1584	0.3744
0.216	0.3312	0.2016	0.8496	0.3888	0.0864
0.3744	0.4608	0.3456	0.36	0.3024	0.1008
0.216	0.1584	0.1728	0.9792	0.2592	0.1008
0.1584	0.4752	0.648	0.1152	0.7056	0.1008
0.2016	0.1008	0.216	0.72	0.5328	0.1152
0.4032	0.288	0.2304	0.1872	0.8784	0.504
0.72	0.2016	0.4896	0.2592	0.504	0.0864
1.0656	0.144	0.6048	0.5184	0.3456	0.2592
0.072	0.3312	0.3312	0.5472	1.008	0.072
0.1872	0.6624	0.2016	0.1296	0.7776	1.008
1.0512	0.6336	0.4608	0.2016	0.72	1.1808
1.2528	0.4032	0.5328	0.864	0.1872	1.1952
0.2736	0.36	0.2592	0.504	0.36	0.6624
0.4464	0.2016	0.3312	0.6336	0.504	0.1872
0.1872	0.4896	0.36	0.4176	0.576	0.2592
0.2304	0.2448	0.216	3.1248	0.72	0.4464
0.288	0.0288	0.3168	1.7136	0.7344	0.3456
0.144	0.288	1.08	0.5184	0.0864	1.0944
0.3888	0.504	0.288	0.8784	0.8784	0.1296
0.4032	0.0864	0.4464	0.3168	0.3456	0.8208
0.144	0.288	0.2448	0.2592	0.1584	0.5904
0.288	1.1232	0.1584	0.1728	0.2016	0.8496
0.1296	0.4464	1.1808	0.4032	0.6624	0.576
0.1872	0.1584	0.2592	0.1872	0.504	1.3104
0.3024	0.2592	0.2448	0.4032	0.1872	0.3744
0.9936	0.864	0.3456	0.3168	0.288	0.4896
0.6192	0.8208	0.1872	0.8208	1.0512	0.1584
1.0944	0.0864	0.2592	0.144	0.3312	0.1152
0.144	0.72	0.648	0.288	0.3312	0.2736
0.4752	0.432	0.6048	1.008	0.4896	0.1728
0.3024	0.6336	0.9648	0.216	0.3456	0.3888
0.1584	0.216	1.008	0.3456	0.072	0.4464
0.072	0.4608	2.0016	0.5328	0.1872	0.3888
0.2304	0.0864	1.2528	0.1296	0.144	0.2592
0.3744	0.4896	0.72	0.3312	0.8784	0.5904
0.072	0.3024	0.9072	0.5328	0.7056	0.5328
0.1296	1.224	0.216	0.1008	0.6624	0.432
0.6048	0.4032	0.5184	0.6624	0.1872	0.6336
0.3312	0.3024	0.3888	1.4832	0.9648	0.5328
0.7488	0.2448	0.4176	0.1008	0.3456	0.144
0.2016	0.072		0.792	0.1152	0.8928
0.144	1.6128		0.6192	0.2304	0.3888
0.1296	0.5616		0.1008	0.3168	0.9216
0.504	0.2736		0.432	0.2016	0.7056
0.1584	0.7488		0.4176	0.216	0.8784
1.512	0.2592		0.9648	0.2448	0.072
0.3744	0.3888		0.3024	0.2736	0.5616
0.9936	0.576		0.0864	0.9072	0.5472
0.1152	0.0864		0.3168	0.216	0.3168
0.6336	0.7632		0.936	0.2736	0.6768
0.3168	0.288		0.1728	0.2736	0.2592
0.3024	0.1872		0.7056	0.6624	0.2304
0.216	0.1152		0.1008	0.36	0.2592
0.5328	0.4176		0.2448	0.7632	0.288
1.0656	0.2736		0.216	0.3456	0.1152
0.2736	0.288		0.6192	0.1152	
0.648	0.3312		0.4032	0.072	
