# Supplementary material for: Defective glutamate and K+ clearance by cortical astrocytes in familial hemiplegic migraine type 2
Source: EMBO Mol Med. 2016 Jun 27;8(8):967–86. doi: 10.15252/emmm.201505944 (PMC4967947; doi:10.15252/emmm.201505944)
Supplement: Supplementary file 13 — Source Data for Figure 9 [file EMMM-8-967-s011.pdf]

Figure 9 Panel A Left Source Data

Time (s)	STC tau decay				
15	5.77	675	6.61198	1335	7.09284
30	5.34	690	6.09742	1350	6.8725
45	5.08458	705	6.87822	1365	7.48795
60	5.42924	720	7.13026	1380	7.03268
75	5.28774	735	7.05758	1395	6.88738
90	5.60628	750	6.96302	1410	7.12741
105	5.69047	765	6.80735	1425	7.0813
120	5.42177	780	6.83916	1440	7.21581
135	5.54785	795	7.3192	1455	--
150	5.15474	810	6.83072	1470	6.6121
165	5.53561	825	6.90493	1485	7.07391
180	5.50552	840	7.04563	1500	7.0132
195	5.04546	855	6.61302	1515	6.93033
210	5.50844	870	6.92988	1530	7.26288
225	5.5792	885	6.84655	1545	6.73957
240	5.46278	900	7.05704	1560	--
255	5.90288	915	6.98436	1575	--
270	5.39092	930	6.79629	1590	6.58
285	5.56718	945	6.53115		
300	5.19311	960	7.00134		
315	5.60837	975	7.12983		
330	5.52656	990	6.99818		
345	5.86955	1005	6.98368		
360	5.40967	1020	6.60699		
375	6.23855	1035	6.618		
390	5.68257	1050	6.7918		
405	6.27592	1065	7.21037		
420	6.04825	1080	6.99016		
435	6.55361	1095	7.48457		
450	6.00263	1110	7.25433		
465	6.37187	1125	7.48794		
480	6.32072	1140	7.2107		
495	5.84317	1155	6.93751		
510	6.13275	1170	7.41675		
525	6.05236	1185	7.24897		
540	6.23371	1200	6.96856		
555	6.01111	1215	6.76536		
570	6.64472	1230	6.95943		
585	6.22965	1245	6.60133		
600	6.49566	1260	7.13955		
615	6.01257	1275	6.917		
630	6.13194	1290	6.66432		
645	6.71725	1305	7.52003		
660	6.58102	1320	6.91767		

Figure 9 Panel A Right Source Data

STC τ_{decay} Ctr	STC τ_{decay} TBOA 2.5
5.09	6.72
7.55	10.08
8.04	10.21
7.02	9.53

Figure 9 Panel B Left Source Data

Ctr Thresh	TBOA 2.5 Thresh
230	150
230	130
290	150
230	150
290	150
190	170
190	150
210	130
190	170
190	170
190	130
210	170
230	170
210	150
230	150
290	130
190	130
190	110
170	150
210	150
190	110
230	150
290	110
	110
	110

Figure 9 Panel B Right Source Data

Ctr velocity	TBOA 2.5 velocity
3.11	3.33
2.75	4.09
2.43	4.07
3.29	3.81
3.41	3.24
3.10	3.3
2.95	3.43
2.63	3.58
3.32	4.29
3.22	3.96
3.16	3.85
3.34	3.48
3.77	3.71
3.05	3.93
3.72	3.98
3.56	3.59
3.31	2.95
3.11	3.9
3.79	4.44
3.57	4.72
2.55	3.08
3.72	4.24
3.66	4.25
2.53	4.48
	4.08
	3.93

Figure 9 Panel C Source Data

STC τ_{decay} Ctr	STC τ_{decay} TBOA 1.5
7.55	8.85
6.12	7.84
6.04	7.01
6.11	7.81

Figure 9 Panel D Left Source Data

Ctr Thresh	TBOA 1.5 Thresh
230	150
230	170
290	150
230	170
290	190
190	190
190	170
210	170
190	190
190	210
190	150
210	170
230	170
210	170
230	190
290	170
190	150
190	130
170	
210	
190	
230	
290	

Figure 9 Panel D Right Source Data

Ctr velocity	TBOA 1.5 velocity
3.11	4.04
2.75	3.82
2.43	3.23
3.29	3.39
3.41	3.87
3.10	3.56
2.95	3.76
2.63	2.99
3.32	3.32
3.22	4.24
3.16	4.16
3.34	3.87
3.77	3.27
3.05	3.72
3.72	4.2
3.56	3.77
3.31	3.18
3.11	3.11
3.79	3.11
3.57	
2.55	
3.72	
3.66	
2.53	
